# Identification of Y-Box Binding Protein 1 As a Core Regulator of MEK/ERK Pathway-Dependent Gene Signatures in Colorectal Cancer Cells

**DOI:** 10.1371/journal.pgen.1001231

**Published:** 2010-12-02

**Authors:** Karsten Jürchott, Ralf-Jürgen Kuban, Till Krech, Nils Blüthgen, Ulrike Stein, Wolfgang Walther, Christian Friese, Szymon M. Kiełbasa, Ute Ungethüm, Per Lund, Thomas Knösel, Wolfgang Kemmner, Markus Morkel, Johannes Fritzmann, Peter M. Schlag, Walter Birchmeier, Tammo Krueger, Silke Sperling, Christine Sers, Hans-Dieter Royer, Hanspeter Herzel, Reinhold Schäfer

**Affiliations:** 1 Laboratory of Molecular Tumor Pathology, Universitätsmedizin Berlin, Berlin, Germany; 2 Laboratory of Functional Genomics, Universitätsmedizin Berlin, Berlin, Germany; 3 Institute for Theoretical Biology, Humboldt University, Berlin, Germany; 4 Max Delbrück Center for Molecular Medicine, Berlin, Germany; 5 Max Planck Institute for Molecular Genetics, Berlin, Germany; 6 Institute of Pathology, Friedrich-Schiller-University Jena, Jena, Germany; 7 Charité Comprehensive Cancer Center, Berlin, Germany; 8 Center of Advanced European Studies and Research, Bonn, Germany; 9 Institute of Human Genetics and Anthropology, Heinrich-Heine University Düsseldorf, Düsseldorf, Germany; University of Pennsylvania, United States of America

## Abstract

Transcriptional signatures are an indispensible source of correlative information on disease-related molecular alterations on a genome-wide level. Numerous candidate genes involved in disease and in factors of predictive, as well as of prognostic, value have been deduced from such molecular portraits, e.g. in cancer. However, mechanistic insights into the regulatory principles governing global transcriptional changes are lagging behind extensive compilations of deregulated genes. To identify regulators of transcriptome alterations, we used an integrated approach combining transcriptional profiling of colorectal cancer cell lines treated with inhibitors targeting the receptor tyrosine kinase (RTK)/RAS/mitogen-activated protein kinase pathway, computational prediction of regulatory elements in promoters of co-regulated genes, chromatin-based and functional cellular assays. We identified commonly co-regulated, proliferation-associated target genes that respond to the MAPK pathway. We recognized E2F and NFY transcription factor binding sites as prevalent motifs in those pathway-responsive genes and confirmed the predicted regulatory role of Y-box binding protein 1 (YBX1) by reporter gene, gel shift, and chromatin immunoprecipitation assays. We also validated the MAPK-dependent gene signature in colorectal cancers and provided evidence for the association of YBX1 with poor prognosis in colorectal cancer patients. This suggests that MEK/ERK-dependent, YBX1-regulated target genes are involved in executing malignant properties.

## Author Summary

The simultaneous analysis of gene expression in cancer using microarrays is a standard approach for monitoring disease-related modifications involved in tumorigenesis, triggering malignant properties and clinical behavior. However, the factors that drive these alterations most often remain elusive. We sought to identify transcription factors that mediate the transcriptional effects of the receptor tyrosine kinase/RAS oncoprotein pathway, a frequently activated oncogenic signaling system, in cultured colorectal cancer cells. We used an integrated approach combining molecular and functional assays, as well as computational tools, to identify regulatory factors that trigger the alterations of gene expression and modulate cellular growth. We identified the YBX1 protein, a member of the highly conserved family of cold shock domain transcription factors, as a regulator of signaling effects triggered by the RAS cancer gene. Then we assayed the messenger RNA expression of YBX1 and YBX1-responsive target genes by interrogating microarrays, and also expression of the YBX1 protein by immunohistochemistry in colorectal tumors. We found that YBX1 expression is correlated with a bad clinical outcome in colon cancer patients.

## Introduction

Transcriptional signatures were established for thousands of cancer specimens and correlated with disease classification, progression, prognosis and therapy response [1]–[3]. While the clinical implications of these data are continuously attracting high attention, the principles of global disease-related gene deregulation and their functional consequences are still poorly understood. A traditional approach for moving correlative gene expression-based information to the functional level is to select one or few individual factors from disease-associated signatures and to study the candidate genes in detail. However, this experimental strategy is not feasible when hundreds of deregulated genes, or even combinations of them, need to be analyzed. Investigations of signaling proteins and other regulatory factors hold great promise, because such factors can control multiple downstream genes and therefore potentially qualify as the major drivers of transcriptional signatures [4]–[6]. Several lines of evidence have suggested that the signaling-mediated transcriptional response ultimately involved in executing cancer phenotypes exhibits a modular organization [7]–[10]. Common elements of these modules are proteins of the signaling network. Transcriptional regulators downstream of the signaling cascades may either be included among the module elements or not be components of the gene signature. To understand the regulatory principles governing cancer-associated gene signatures, a detailed analysis of such modules is needed.

The receptor tyrosine kinase (RTK)/RAS pathway serves as a paradigmatic example for studying the functional and regulatory properties of oncogenic signaling networks and their targets. Many RTK-mediated signals converge on RAS proteins as major molecular switches for linking cytoplasmic signal transduction with the underlying genetic program [11]. The RTK/RAS pathway triggers multiple properties of cancer cells [12]; [13]. At the phenotypic level, downstream signaling pathways activated by RAS elicit cell type-specific, but also overlapping effects such as proliferation, cellular survival and transformation [14]–[16]. RAS-related gene expression profiles have been described in various cellular models of malignant transformation [7]; [10]; [17]. More recently, the clinical relevance of RAS research has been highlighted by the finding that KRAS mutations cause resistance to therapies targeting membrane-bound RTKs [18].

Our previous work aimed at cataloguing RAS-responsive target genes in RAS-transformed fibroblasts and epithelial cells. By pathway interference using signaling kinase inhibitors, we identified subsets of target genes (signal-regulated transcriptional modules) responding to two of the major effector pathways downstream of RAS, the BRAF/MEK/ERK(MAPK) pathway and the phosphatidylinositol-3 kinase (PI3K) pathway as well as subsets of target genes not responding to either pathway [8]. We assumed that narrowing down the entire gene expression profile to pathway branch-restricted co-expressed groups of target genes would be an efficient strategy for identifying regulatory factors downstream of the signaling cascade. Therefore, we decided to screen MEK/ERK pathway-controlled transcriptional targets for common cis-regulatory elements. Computational prediction of transcription factor binding sites in cis-regulatory elements of signature genes has been successfully used for a global analysis of factors mediating immediate-early and delayed transcriptional responses during transition from the quiescent to the growth factor-stimulated state [4]; [5].

To identify transcription factors downstream of the MEK/ERK pathway, we chose colorectal cancer cell lines as a model rather than generic cell lines transfected with *RAS* genes that exhibit artificially high RAS protein levels. The cell lines harbor endogenous KRAS or BRAF mutations which drive tumorigenesis in the colon in concert with further typical genetic alterations in *APC* and *TP53* genes [19]; [20]. We analyzed non-synchronized cells in logarithmic growth phase to avoid extensive overlap with gene signatures characteristic for growth factor-stimulated transition from the quiescent to the proliferative state and to mimic the conditions of cancer cells in various phases of the cell cycle.

In the first step of an integrated analysis, we screened for responsive target genes in the colorectal cancer cell lines treated with several inhibitors of the RTK/RAS/RAF/MEK pathway. Once different sets of pathway-dependent genes were identified, we subjected the cis-regulatory sequences of clustered target genes to *in silico* analysis of potential transcription factor binding sites. In the second step, we provided biochemical evidence for the specific role of one of the predicted factors, Y-box binding protein 1 (YBX1), in controlling a significant part of the MEK/ERK(MAPK)-dependent transcription of proliferation-associated genes. Finally, we investigated the MAPK signature and the role of YBX1 as a prognostic factor in primary and metastatic colorectal cancers.

## Results

### Identification of MEK/ERK-dependent targets in colorectal cancer cells

**Figure 1:**
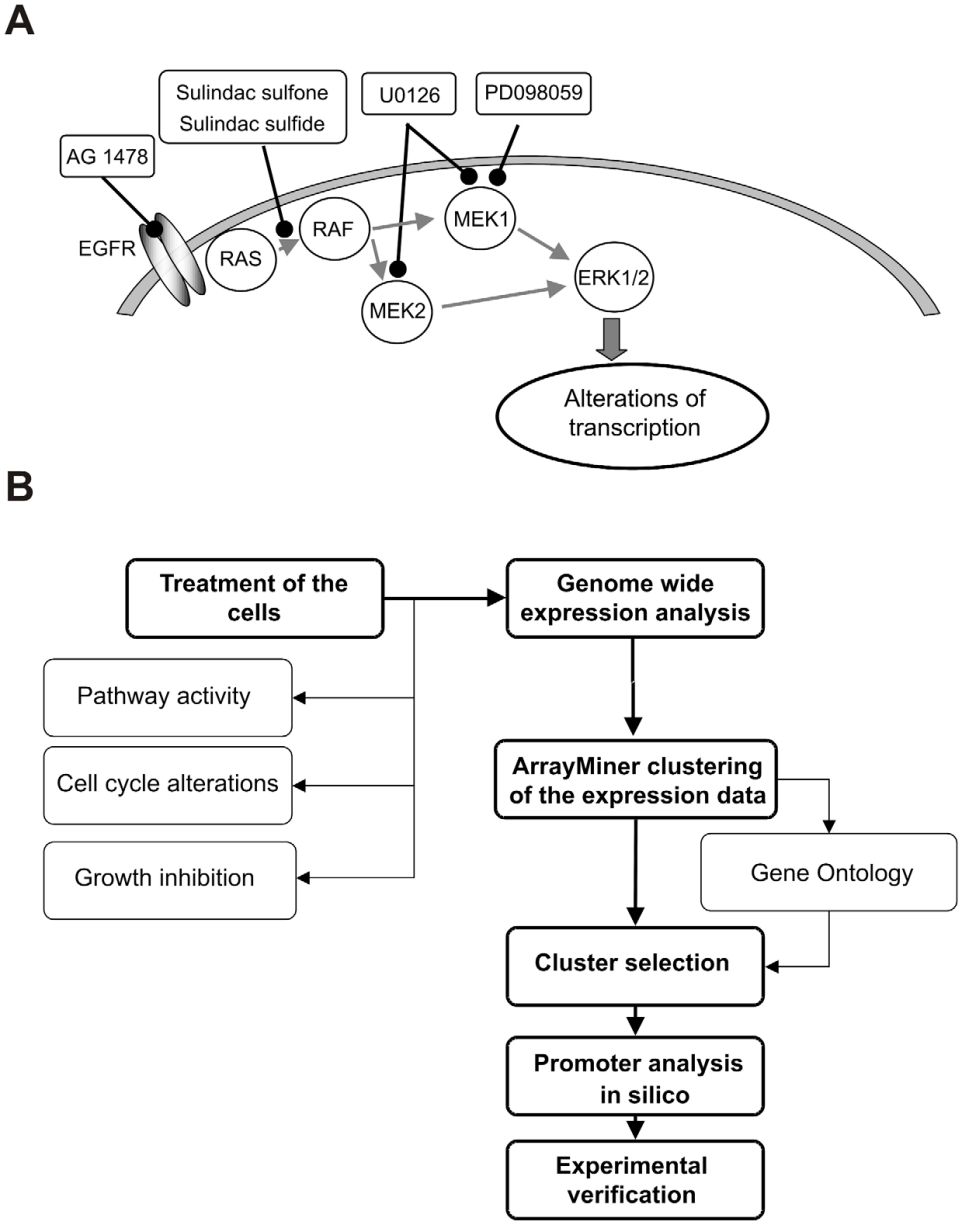
Outline of the experimental design. Outline of the experimental design for exploring the effects of individual elements of the RTK/RAS/MEK/ERK signaling pathway on the global transcription pattern in colon carcinoma cells. (A) Components of the signaling pathway and their modulation by inhibitors. Arrows indicate activation, ---o, inhibition. (B) Flow chart of wet lab and computational analysis.

The transcriptional program in colorectal cancer cells is profoundly affected by genetic alterations in cellular signaling systems such as WNT/APC/β-catenin, epidermal growth factor receptor (EGFR)/RAS/MEK/ERK and DPC4/SMAD4/TGF-β pathways as well as in transcriptional regulators such as TP53 [20]. Therefore, the identification of transcriptional changes related to RAS/MAPK signaling required a strategy for separating the specific from non-MEK/ERK-driven effects. We used an approach combining expression profiling of three different tumor cell lines, reflecting the typical genetic background of primary colorectal cancers, and pathway interference by inhibitors targeting different elements of the RTK/RAS/MEK/ERK signal cascade (Figure 1). SW480 cells harbor a mutation at codon 12 of the *KRAS* gene, mutated *TP53* and *APC* tumor suppressor genes. HCT116 cells carry a mutation at codon 13 of *KRAS*, a *CTNNB1* mutation, wild-type *TP53* and *APC* genes. HT29 cells express wild-type *KRAS*, and mutated BRAF, *TP53* and *APC* genes [21]; [22]. To perturb the signaling pathway, we used the EGFR inhibitor AG1478 [23], two sulindac metabolites, sulindac sulfide and sulfone known to block RAS/RAF interaction besides their canonical function as cyclooxygenase-2 inhibitors [24], and the MEK inhibitors PD098059 and U0126 [25]; [26]. The duration of treatments was at least 48 h to allow sufficient time for monitoring effects on cellular growth and survival.

**Figure 2:**
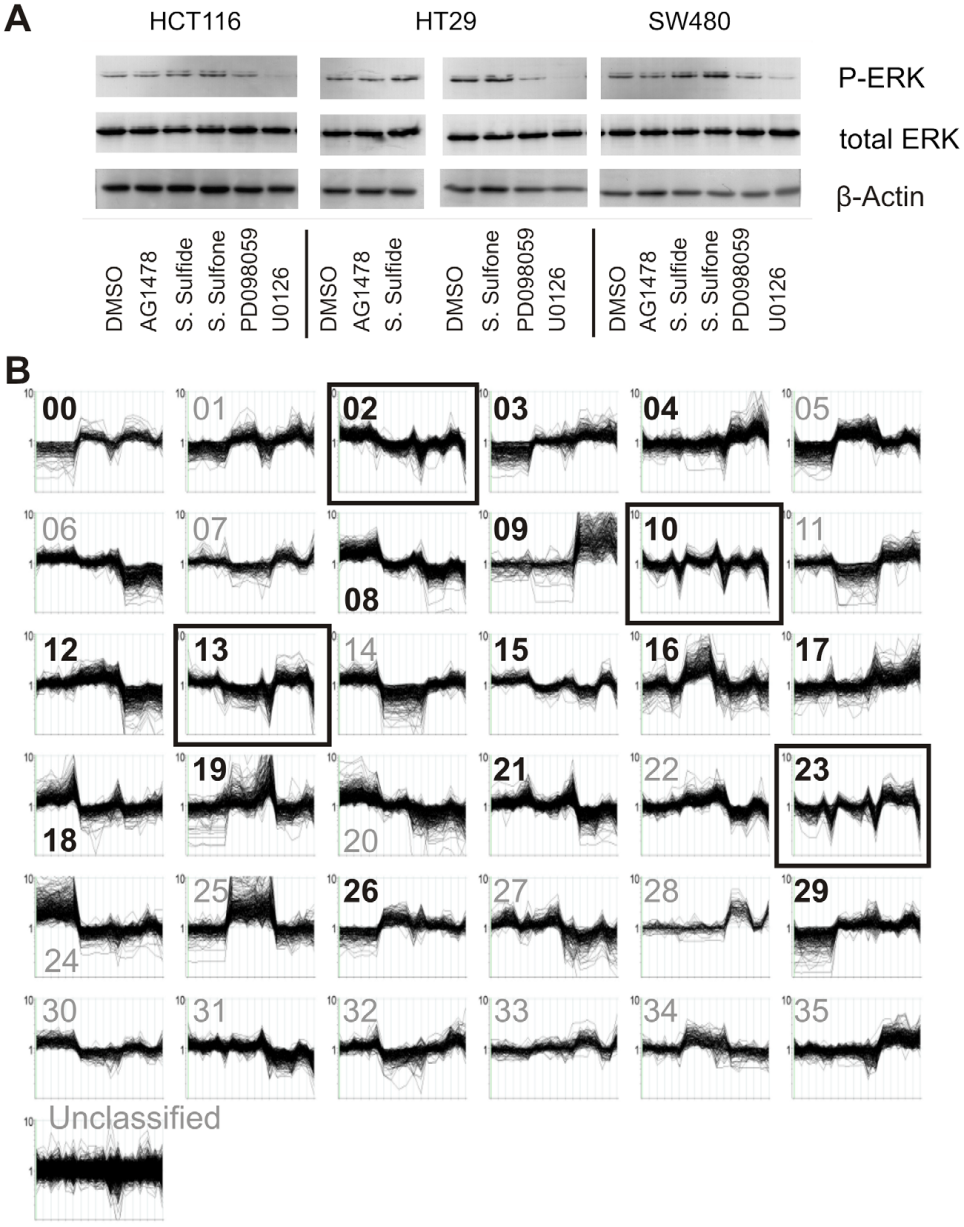
Effects of MEK-inhibition on downstream signaling and transcriptomic profiles. (A) Western blot analysis of phosphorylated ERK levels in colon carcinoma cells treated with the indicated inhibitors for 48 h. DMSO, solvent-only control. Total ERK and β-actin levels were determined to control for equal loading of cellular lysates. (B) Microarray data (number of target RNA samples  = 18) were clustered using the ArrayMiner algorithm and 36 clusters of co-expressed genes were defined. The numbering of clusters starts with cluster 0 and ends with cluster 35. The ordinates of each cluster graph represent hybridization intensities on a logarithmic scale. The abscissas of each cluster graph depict the cell lines and treatments in the same order as in A (from left to right): HCT116 cells treated with DMSO (solvent control), AG1478, sulindac sulfide, sulinac sulfone, PD98059, and U0126; HT29 cells, treated with the same compounds and SW480 cells, treated in the same way (totaling 3 cell lines with 6 treatments each). Genes in clusters marked by black boxes exhibit strong down-regulation of expression after U0126 treatment and an over-representation of gene ontology (GO) terms related to cell cycle regulation, indicating a link between this process and transcriptional control (Table S3). Genes in clusters marked by bold numbers show over-representation of at least one of further GO terms listed in Table S4. Some of the clusters reflect cell line-specific differences in gene expression, e.g. genes grouped in clusters 04, 09 were more abundantly expressed in SW480 than in the two other cell lines, independent of inhibitor treatment. Cluster 04 transcripts encode proteasome components, suggesting a difference in ubiquitin-dependent protein catabolism between SW480 and other cells. We also detected an overrepresentation of genes associated with development and morphogenesis in cluster 09. HT29 and HCT116 cells exhibit an epithelial morphology and grow in compact colonies, while SW480 cells show a spindle-like shape and grow in a scattered fashion until they reach confluence (data not shown). It is likely that cluster 09 genes contribute to these morphological differences.

To assess the inhibitor effects on the signaling network, we determined the phosphorylation status of c-RAF, MEK1/2 and ERK 1/2. The MEK inhibitor U0126 completely abolished the phosphorylation of ERK1/2 in all cell lines (Figure 2A), while the response of the signaling network to the other inhibitors was more heterogeneous (Figure S1A). These effects ranged from partial inhibition of the down-stream kinases to unexpected up-regulation of phosphorylation possibly due to cross-talk or feed-back mechanisms triggered by transient pathway interference. We did not observe inhibition of PKB/AKT phosphorylation at Ser^473^, indicating that the PI3-kinase pathway was unaffected (Figure S1B).

We then contrasted the transcriptional profiles of the three cell lines, treated with each of the five inhibitors separately and a solvent control by interrogating high-density oligonucleotide arrays (Affymetrix HG-U133A). To identify groups of co-expressed target genes affected through pathway inhibition, we subjected 7,049 genes (corresponding to 9,272 probe sets exhibiting significant hybridization intensities) to ArrayMiner clustering. This algorithm identified 36 clusters of co-regulated genes in the entire set of pathway interference experiments (Figure 2B, Tables S1 and S2). Some of the gene clusters reflect cell line-specific responses dependent on signaling interference (e.g. clusters 07, 16, 28) and effects independent of pathway inhibition (e.g. clusters 05, 25). However, consistent alterations of gene expression associated with pathway inhibition were recognized despite the overall transcriptional heterogeneity of the cell lines. Genes down-regulated on treatment with the MEK inhibitor U0126 represent the largest set of responsive targets (n = 776) common to all cells (clusters 02, 10, 13, 23; Figure 2B, Table S3). This indicates that the expression of these genes is sensitive towards MEK/ERK1/2 signaling and, hence, consistently regulated by this pathway. A Gene Ontology (GO) analysis of MEK/ERK1/2 target genes revealed a statistically significant over-representation of functions associated with cell cycle control (Table S4).

**Figure 3:**
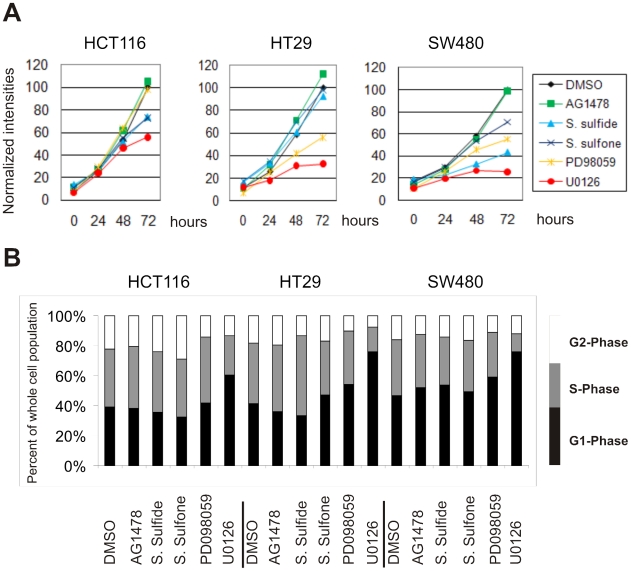
Effects of MEK-inhibition on cell growth and cell cycle progression. (A) Cell growth determined by XTT-based colorimetric assay at 0, 24, 42 and 72 h after adding inhibitors. The mean values for DMSO (control) at 72 hours were set to 100 and all other values were normalized relative to these values. (B) Flow cytometric analysis of the cell cycle distribution in inhibitor-treated colon carcinoma cells. Black bars, cells in G1 phase; grey bars, S-phase; white bars, G2 phase.

The decisive role of the RAS signaling pathway in triggering growth, altered differentiation, progression and therapy resistance of colorectal cancer cells is well established [27]; [28]. As expected, treatment with U0126 strongly inhibited growth, mainly due to blocking the cell cycle in G1 phase (Figure 3A, 3B). Cell cycle analysis did not reveal any sub-G1 peaks following inhibitor treatment and we did not observe any morphological changes indicative of cell death. Thus, apoptosis was excluded as the cause for reduced cell growth. The other inhibitors exhibited less consistent effects. PD098059 treatment, sufficient for blocking MEK1 (IC_50_ = 4 µM) but not MEK2 (IC_50_ = 50 µM), reduced growth of HT29 and SW480, but did not significantly affect HCT116 cells. Sulindac sulfide and sulfone inhibited growth of HCT116 and SW480, but not of HT29 cells. This supports the notion that proliferation in HT29 cells is driven by the BRAF mutation and is insensitive to perturbing the RAS/RAF interaction. AG1478 treatment had no effect, suggesting that KRAS or BRAF mutations are sufficient for triggering growth and downstream signaling effects.

### Identification of overrepresented transcription factor binding sites in genes regulated by the MEK/ERK pathway

**Figure 4:**
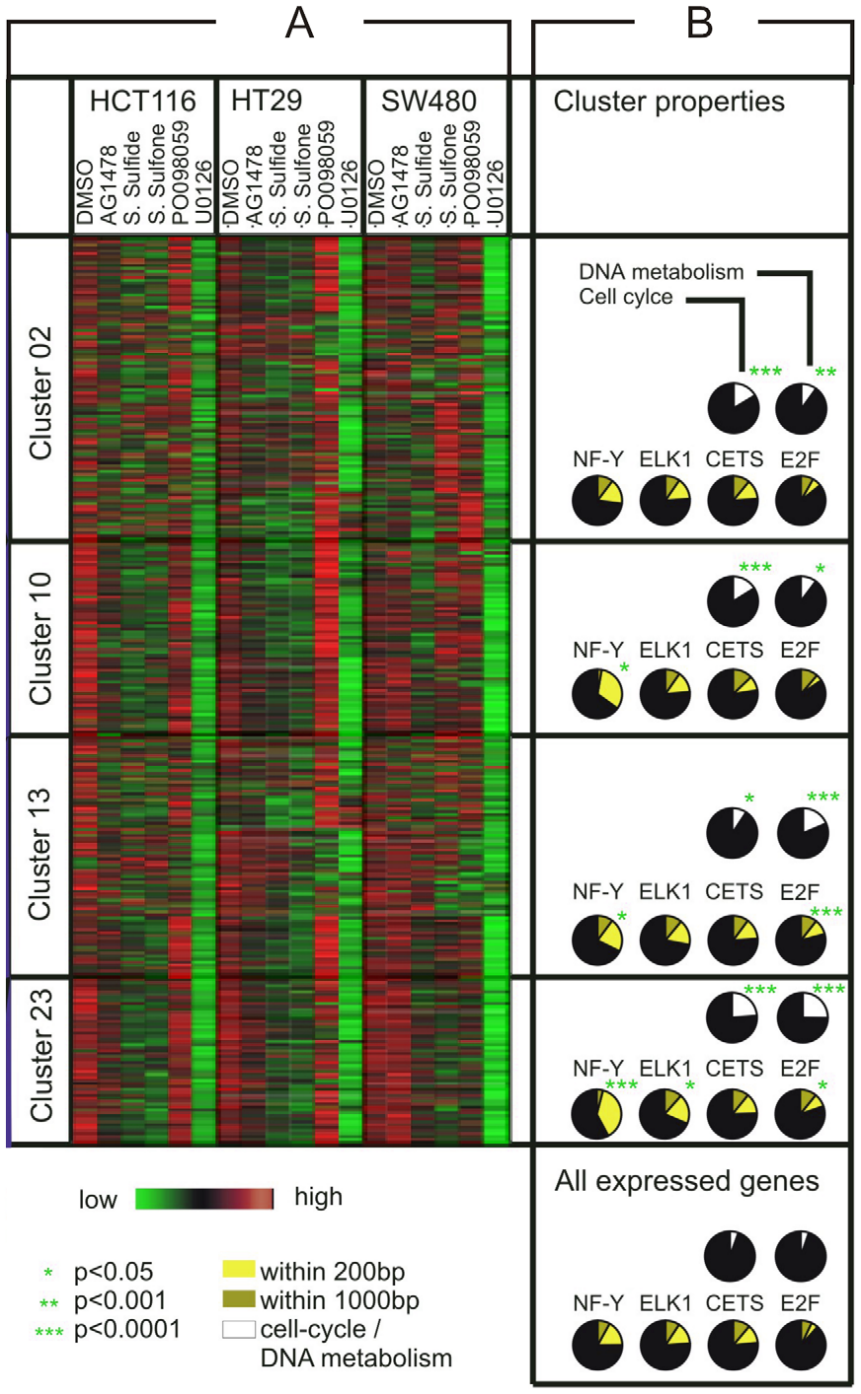
Predicted biological function and transcription factor binding sites of MEK/ERK pathway-dependent target genes in colon cancer cells. (A) Heat map of co-regulated genes (clusters 02, 10, 13, 23) in HCT116, HT29 and SW480 cells. Expression values were standardized separately for each cell line to demonstrate the common pattern of target gene down-regulation after blocking the pathway with the MEK inhibitor U0126. Green, reduced expression; red, high expression. (B) Fraction of genes attributed to the GO terms “Cell cycle” and “DNA metabolism” in clusters (white sectors in black circles). Prevalence of transcription binding factor binding motifs for NFY, ELK1, CETS and E2F within gene promoters at a distance of 200-bp (light yellow sectors in black circles) and 1000-bp (dark yellow sectors) from the transcription start site, respectively. The statistical significance (p-value) is indicated by asterisks.

To elucidate the potential mechanisms underlying the coordinated expression of MEK/ERK pathway-regulated genes in colorectal cancer cells, we screened the predicted promoter regions (1,000 nucleotide sequences upstream of the transcriptional start site of each gene) of all 7,047 informative genes for 559 known transcription factor binding sites assembled in TRANSFAC. We compared the number of predicted binding sites in the promoters of 776 MEK/ERK-dependent genes with their overall abundance in the entire promoter set. To limit the extent of false predictions, we used a strict multiple testing framework [29]. As a result, E2F, NFY and HOXA4 transcription factor binding motifs occurred significantly more often than expected by chance (Table S5). The matrix identifiers were E2F1_Q4_01, E2F_Q3_01, E2F_Q4_01, NFY_01, HOX_A4 and the false discovery rate was <0.05. Subsequently, we screened the promoter sequences of the clustered genes separately for overrepresentation of E2F and NFY binding-motifs. In addition, we investigated the target sequences of the known MAPK-regulated transcription factors c-ETS and ELK. The matrix identifiers were E2F1_Q4_01, NFY_01, CETS1P54_01, and ELK1_02, respectively. E2F and NFY binding sites were strongly overrepresented in target gene clusters 13 and 23, and NFY motifs solely in cluster 10. C-ETS and ELK binding motifs were less prevalent, except for ELK1 motifs in cluster 23. The predicted functional relevance of the recovered NFY and E2F binding sites in gene clusters 10, 13 and 23 was further supported by their close proximity (200-bp upstream) to the transcription start sites of MEK/ERK-dependent genes (Figure 4).

**Figure 5:**
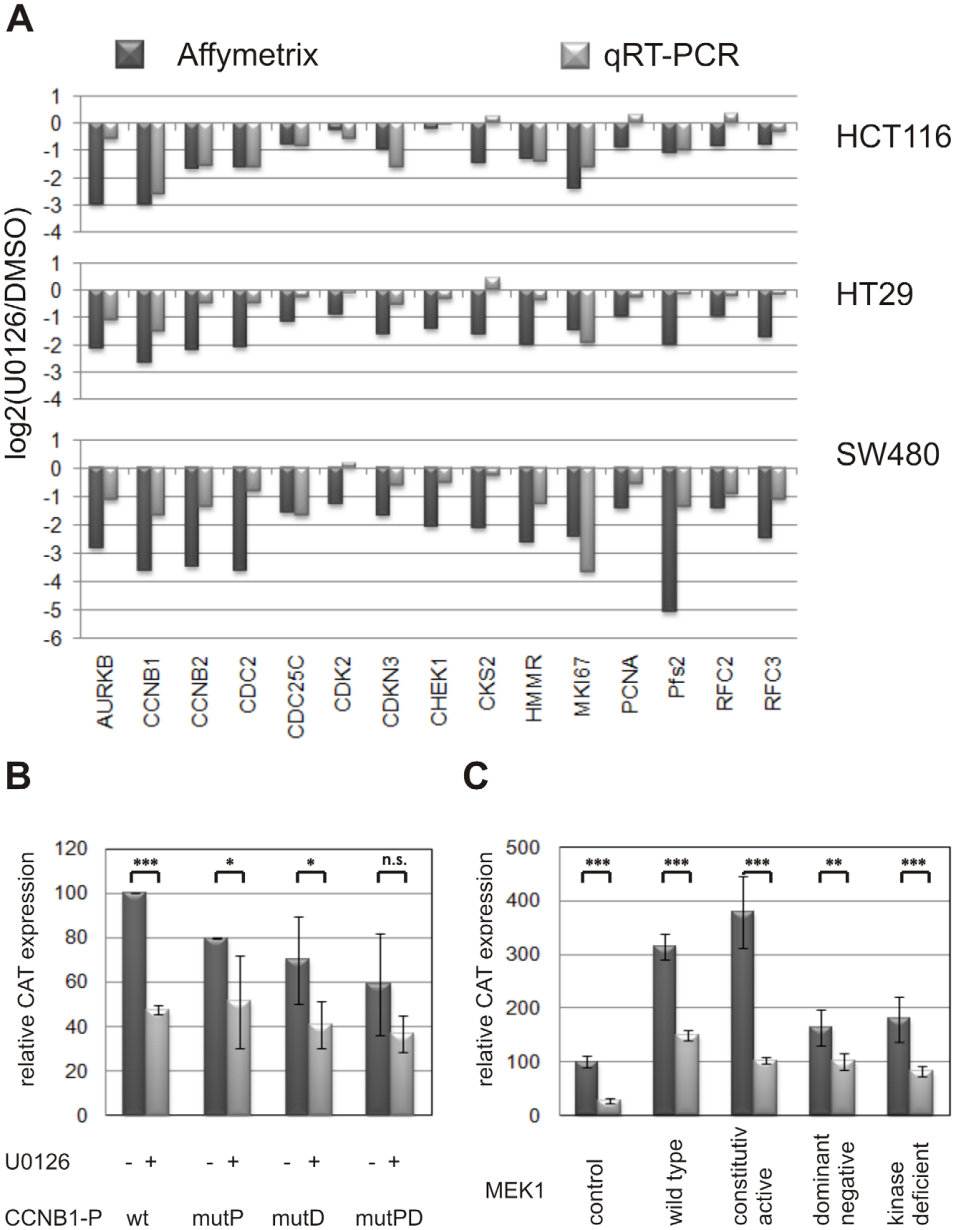
Role of NFY binding sites in MEK/ERK-dependent gene regulation. (A) Validation of microarray-based expression data (black bars) by quantitative real-time PCR on TaqMan low density arrays (grey bars). Gene expression data are shown as log2 ratios of RNAs/cDNAs prepared from HCT116, HT29 and SW480 cells treated with U0126 for 48 hours and DMSO (solvent) controls. (B) *CCNB1* promoter activity in transiently transfected HCT116 cells. The activity of the −57 to +182 bp promoter fragment harboring 2 NFY-binding sites (p240-wt) and controlling the expression of the chloramphenicol acetylase (CAT) reporter was set to 100%. Light bars indicate CAT expression after U0126 treatment for 48 h, dark bars represent DMSO (solvent)-treated controls. Mutated promoters: p240-mP, mutation of proximal NFY-site; p240-mD, mutation of the distal NFY-site; p240-mPD, double mutant. (C) *CCNB1* promoter activity in HEK293 transfectants stably expressing wild-type, constitutively active, dominant negative and kinase-deficient MEK1 gene constructs, respectively. Activities of the wild-type (p240-wt; dark bars) and mutated (p240-mPD; light bars) *CCNB1* promoter:CAT reporters were determined after transient transfection of MEK gene constructs.

While the role of E2F transcription factors in RAS-dependent signal transduction and transformation is well established [30], our results suggested an important functional relationship between RAS/MEK/ERK signaling and transcription factor binding to NFY sites. The core motif RRCCAATSRG is a frequent regulatory element in eukaryotic promoters and is operative in forward (CCAAT-box) or reverse orientation (Y-box). To confirm the functional role of NFY sites in the regulation of proliferation-associated genes within the clusters, we chose the cyclin B1 (*CCNB1*) promoter as a model. The CCNB1 protein is a central regulator of the transition from G2 phase to mitosis. *CCNB1* belongs to proliferation-associated cluster 23 (Figure 4). Its promoter comprises two NFY-binding sites [31]. We verified *CCNB1* mRNA down-regulation following U0126-treatment independently in all colon carcinoma cell lines (Figure 5A) and determined *CCNB1* promoter activity in transiently transfected HCT116 cells using a chloramphenicol acetyltransferase (CAT) reporter gene controlled by the 240-bp promoter fragment harboring the two NFY-binding sites [32]. U0126 treatment of HCT116 cells reduced promoter activity to a basal level of 50% (p-value: <0.00025, single-sided t-test). The basal activity was insensitive to MEK inhibition. We observed an equal reduction of promoter activity using promoter constructs with the two inactive NFY-binding sites (Figure 5B). Although reporter activity appeared to be further diminished in cells transfected with the mutated *CCNB1* promoter and treated with U0126, the difference did not reach statistical significance (p-value: >0.05). Therefore, MEK inhibition and elimination of NFY binding sites were not synergistic, indicating that both manipulations mainly affect the identical mechanism.

To further analyze the influence of upstream MEK signaling on *CCNB1* expression, we stably transfected various *MEK* expression constructs into HEK293 cells expressing wild-type RAS. Subsequently, *CCNB1* promoter activity was determined by transient transfection of the reporter constructs. Wild-type *CCNB1* promoter activity was strongly enhanced in cells expressing wild-type or constitutively active *MEK1*, while the activity of the mutated promoter did not exceed the basal level (Figure 5C). These results further supported the critical role of NFY binding sites for MEK-dependent regulation of *CCNB1* mRNA expression.

### YBX1 mediates transcriptional effects of RAS/MEK/ERK-signaling

**Figure 6:**
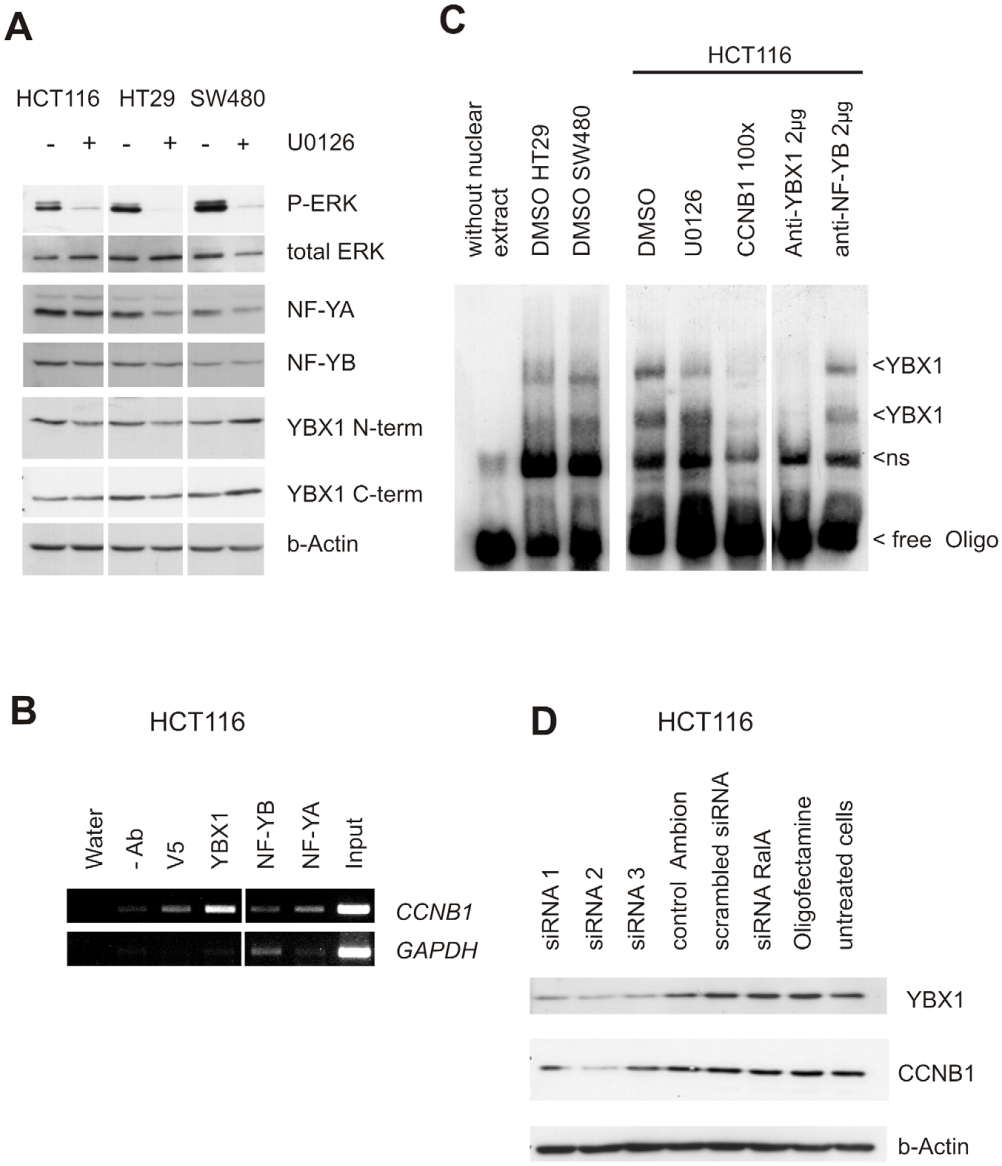
Regulation of CCNB1 expression by YBX1. (A) Western blot analysis of YBX1 and NFY protein levels in cells treated with the MEK inhibitor U0126 and controls. 20 µg of whole cell lysates were loaded per lane. The indicated antigens were detected. β-actin was used as a loading control. (B) Analysis of NFY binding sites of the *CCNB1* promoter by chromatin-immunoprecipitation using YB-1, NF-YB and NFYA antibodies. Immune complexes were prepared from sonicated nuclear extracts prepared from HCT116 cells and PCR-amplification of bound DNA as described in Methods. A fragment of the GAPDH promoter was used as control. (C) Binding of YBX1 to the *CCNB1* promoter analyzed by gel retardation assay. Arrows indicate the specific retardation of the 60-bp *CCNB1* promoter fragment (ns, non-specific binding). Nuclear extracts were prepared from HT29, SW480 (left) and HCT116 cells (right). To assess the dependence on MEK/ERK signaling of YBX1 binding to the *CCNB1* promoter, cells were treated with the MEK inhibitor U0126 and DMSO (solvent control) prior to preparing nuclear extracts. To prove the specificity of binding, we performed the gel retardation assay with a 100-fold excess of the unlabeled *CCNB1* promoter fragment (*CCNB1* 100x), and in the presence of YBX1 and NFYB antibodies, respectively. (D) Co-silencing of YBX1 and *CCNB1* protein expression in HCT116 cells as revealed by RNA interference. Three independent siRNAs (YBX1 siRNA1, 2 and 3) targeting YBX1 and scrambled siRNA were transiently transfected. YBX1 protein levels and target *CCNB1* expression were detected by western blotting. β-actin was used as a loading control.

Since microarray and promoter analysis had suggested a role of the MAPK pathway and NFY transcription factors in regulating *CCNB1* expression and activity, we decided to further specify transcription factor:DNA binding. NFY and YBX1 proteins interact with the NFY-motif [33]; [34]. SW480, HT29 and HCT116 cells express YBX1, NFYA and NFYB. U0126 treatment did not consistently alter NFYA, NFYB and YBX1 protein levels (Figure 6A). To find out if any of these factors preferentially bind to the NFY-element in the *CCNB1* promoter, we performed chromatin immunoprecipitation assays using nuclear extracts prepared from HCT116 cells and antibodies specific for YBX1, NFYB and NFYA, respectively. Precipitated DNA was recovered from immune complexes and subjected to PCR amplification using primers specific for the 240-bp *CCNB1* promoter fragment. The endogenous YBX1 protein preferentially binds to the *CCNB1* promoter in nuclear extracts prepared from all colorectal cancer cell lines, while we obtained no evidence for binding of NFYA and NFYB, respectively (Figure 6B).

We confirmed the interaction of YBX1 and the distal NFY-site in the *CCNB1* model promoter by electrophoretic mobility shift assay (Figure 6C). Two specific YBX1:DNA complexes were detected in nuclear extracts using a 60-mer oligonucleotide spanning the NFY-site as binding probe. Pre-incubation with the N-terminal YBX1-specific antibody prevented complex formation, while the NFYB-antibody had no effect. Electrophoretic mobility shifts were efficiently competed by excess of the unlabelled oligonucleotide. Moreover, YBX1:*CCNB1* promoter complex formation was sensitive to U0126 treatment as shown in nuclear extracts prepared from HCT116 cells.

YBX1 was previously described as a transcriptional regulator of *CCNB1* in breast cancer cells and in multiple myelomas [35]. However, YBX1 was not linked to *CCNB1* expression in other cell systems, suggesting a tissue-specific function [36]. To test the impact of YBX1 on *CCNB1* expression in colorectal cancer cells, we transiently silenced YBX1 expression in HCT116 cells by RNA interference. The knock-down of *YBX1* reduced *CCNB1* expression, confirming the role of YBX1 as a *CCNB1* regulator in these cells (Figure 6D). Moreover, silencing of *YBX1* resulted in reduced cell growth, mainly due to a block or a prolongation in G1-phase. Growth reduction was similar to that of the effects of the MEK inhibitor U0126 (Figure S2).

**Figure 7:**
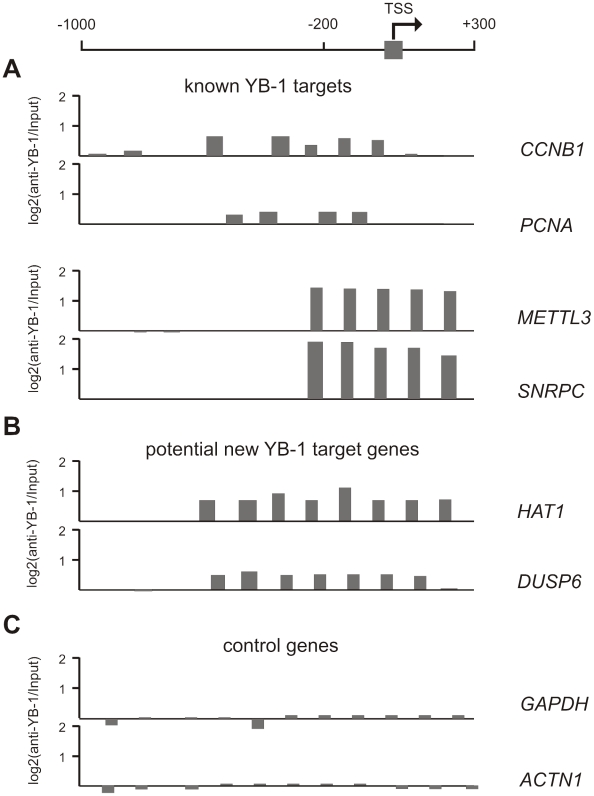
Genome-wide analysis of YBX1:DNA binding by chromatin-immunoprecipitation using NimbleGen ChIP-chip microarrays. The graph on top of the figure represents a schematic illustration of a promoter region (TSS – transcription start side). Extracts of HCT116 cells were used for ChIP-on-Chip analysis with specific antibodies directed against the C-terminus of YBX1. The Y-axis represents the ratio of hybridization intensities (on a log2 scale) of YBX1-precipitated DNA and input DNA. Size and number of the bars on the x-axis depict the enrichment of precipitated DNA fragments around the potential transcription start sites of known (A) and new (B) potential YBX1-target genes. (C) Non-target promoters. *METTL3* (methyltransferase like 3) and *SNRPC* (small nuclear ribonucleoprotein polypeptide C) were recently identified as potential YBX1 target genes in basal-like breast cancer cells by ChIP-on-Chip assay [37]. (*PCNA*: proliferating cell nuclear antigen; *HAT1*: histone acetyltransferase 1; *DUSP6:* dual specificity phosphatase 6; *GAPDH:* glyceraldehyde-3-phosphate dehydrogenase; *ACTN1:* actinin, alpha 1).

To assess the global role of YBX1 as a transcriptional regulator of MEK/ERK-dependent target genes, we prepared YBX1:chromatin immune complexes from HCT116 cells and interrogated genome-wide promoter tiling arrays (NimbleGen Homo sapiens HG17 promoter microarray) using the precipitated DNA fragments as target. We identified DNA fragments enriched by YBX1-antibody precipitation related to 88 genes of the proliferation- associated clusters (Table 1, Figure 7). In agreement with the finding that NFY-binding sites were overrepresented in the regulatory regions of genes in clusters 10, 13 and 23, we found a significant overrepresentation of YBX1-ChIP targets in the same clusters (p-value: <0.02, Fisher's exact test). Twenty-six of them were recently described as potential YBX1 targets in basal-like breast cancer cells as well [37] (Table 1). Promoter sequences of two non-YBX1 target genes, *GAPDH* and *LMNA* were not enriched in YBX1:chromatin immune complexes (Figure 7D).

**Table 1:** Binding of YBX1 to regulatory sequences of MEK/ERK pathway-dependent target genes.

Gene symbol	fold enrichment (smoothed)	Gene symbol	fold enrichment (smoothed)	Gene symbol	fold enrichment (smoothed)
**ACYP1** *	1.5	CDC2 *	1.4	KNTC1 *	2.4
**ADH5** *	1.4	CDC2L2	1.5	LPIN1	1.9
**ASRGL1**	1.7	CGI-01	2.0	MRS2L	1.7
**BMS1L** *	2.1	CSNK2A1 *	2.0	NHP2L1	1.3
**CCNB1** *	1.6	DC12	2.3	NOLC1	1.5
**CDK2**	1.4	DLEU2	1.6	NSL1	1.3
**C14orf156**	1.7	DUSP6	1.5	NTAN1	1.6
**CREM** *	1.8	E1B-AP5	1.7	NXT2 *	1.9
**DLG7** *	1.6	ENY2	1.5	OAZ1 *	1.4
**FKBP3**	1.6	ERH *	1.5	OK/SW-cl.56	1.4
**FTSJ3**	1.4	FAM98A	2.0	PCNT1	2.1
**KIAA0101** *	1.8	FANCG	1.5	PLK4 *	2.1
**KIF2C**	1.4	FLJ12525	1.4	PMSCL1	1.8
**KIFC1** *	1.5	FLJ13912	1.4	PTMA	1.9
**METTL3**	2.7	FLJ14753	1.5	RACGAP1	1.9
**MKI67** *	1.4	FLJ20397	1.6	RCD-8	1.2
**MRPL24**	3.6	FLJ20399	1.5	RNPS1	1.7
**MRPS18A**	1.9	FLJ20516	1.8	SEPHS1	2.0
**NASP** *	1.5	GCN1L1	1.8	SLC39A8 *	1.9
**PHF17**	1.8	H3F3B *	1.6	SHQ1	1.6
**PMVK**	1.3	HAN11	3.0	SUPT16H	1.4
**PREI3** *	1.4	HNRNPA3	1.5	TMEM194A	1.6
**RPA2** *	1.8	HAT1 *	2.1	TTK	1.5
**PRPSAP2** *	2.0	HMGN2	1.4	UBAP2L *	1.8
**SNRPC**	3.7	HPRP8BP	1.9	USP10 *	1.6
**TIMM8B**	2.0	HSPA8 *	1.5	WBP11	1.7
BIRC5	1.7	HUMGT198A	1.7	ZC3H14	1.5
BMP2K	1.7	KIAA1018	1.5	ZNF207 *	1.6
BUB1B *	1.6	KIAA1393	1.4		
C14orf143	2.3	KNSL7	1.4		

88 genes initially identified in proliferation-associated clusters were recovered as YBX1 targets by ChIP-on-Chip assay. Twenty-six of them (depicted in bold face) were also recognized as YBX1 targets in basal-like breast cancer cells [37]. In contrast, only few of the genes in the proliferation-associated clusters were identified by ChIP-on-Chip assay in HepG2 hepatoma cells (PGRMC1, SLC39A8, RFC3) and in the acute lymphoblastic leukemia line Nalm-6 (RAMP, KIAA0101, PCNA, CENPF) [81], suggesting a specific role of YBX1 in breast and colorectal cells.

* Genes which show a significant positive correlation to YBX1 expression in colorectal cancers.

### Transcriptional control through YBX1 is linked to malignant proliferation in colorectal cancer

**Figure 8:**
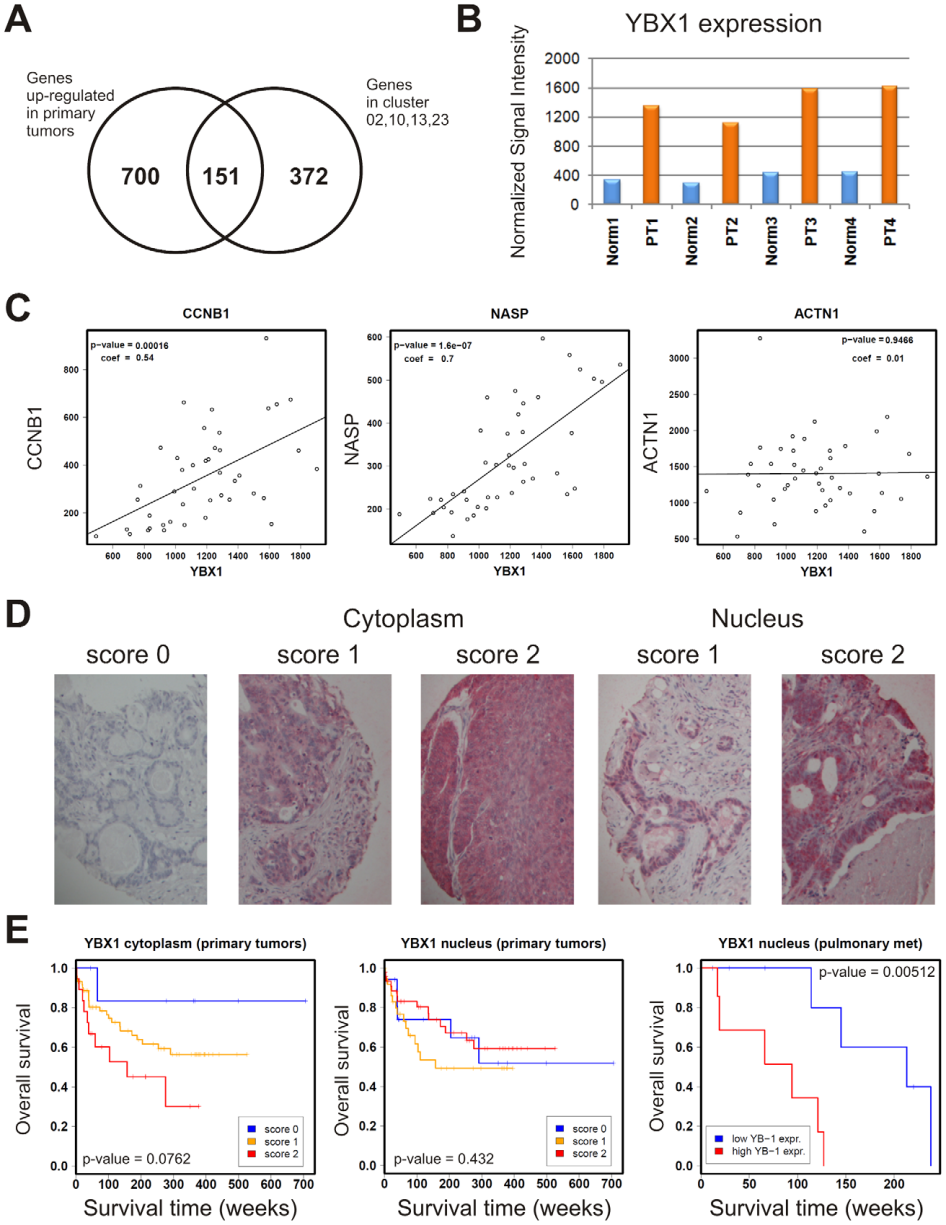
MEK/ERK pathway signature, YBX1 expression, and prognostic value in primary and metastatic colorectal cancer cells. (A) Venn diagram showing the overlap between genes differentially expressed in primary colorectal tumors and MEK/ERK pathway-dependent, proliferation-associated target genes identified in colorectal cancer cell lines treated with the MEK inhibitor U0126. The number of the U0126-responsive genes (523) is smaller than originally identified in the cell lines (776), because there are fewer genes present on the HG-U95A chip compared to the HG-U133A chip. (B) YBX1 expression in matched pairs of colorectal cancers and normal tissues as detected on Affymetrix HG-U95A microarrays. (C) Examples of co-expression of YBX1 and YBX1 targets *CCNB1* and *NASP* (nuclear autoantigenic sperm protein) in a set of 43 primary colorectal tumors as detected on Affymetrix HG-U95A microarrays. Discordant expression of YBX1 and non-target ACTN1 (actinin, alpha 1) shown in the same tumor set. Pearson correlation coefficients (coef) and p-values are inserted in the graphs. Further examples of YBX1 targets are shown in Figure S4. (D) Expression of YBX1 in colorectal tumor sections assembled on tissue microarrays. TMAs were analyzed by immunohistochemical staining using peptide-specific antibodies directed against the N-terminus of YBX1. Cytoplasmic and nuclear staining was scored independently. Score 0, negative for YBX1 expression, score 1, intermediate expression; score 2, high expression. (E) Kaplan-Meier plots showing the association between YBX1 expression and overall patient survival. Left: predominant cytoplasmic YBX1 expression in primary tumors; middle: nuclear YBX1 expression in primary tumors; right: nuclear YBX1 expression in pulmonary metastasis. Clinical data are shown in Table S7.

After identifying target genes of MEK/ERK signaling and assessing the role of YBX1 in colorectal cancer cell lines, we analyzed the clinical relevance in colorectal carcinomas. First, we compared the gene expression profiles of four primary colorectal tumors, in which YBX1 is up-regulated (Figure 8), with their matched normal tissues [38]. We identified 851 genes overexpressed and 311 genes underexpressed at least two-fold in tumors relative to their normal controls (Figure S3, Table S6). We found 151 genes of the proliferation-associated clusters, defined in colorectal cell lines, to be overexpressed in the tumors (Figure 8A). None of them were underexpressed.

Next we investigated the relationship between YBX1 expression and the MEK-dependent, proliferation-associated gene signature in a larger set of 43 primary colorectal cancers. Overall, the expression of about 48% of the genes identified in proliferation- associated clusters was positively correlated with YBX1 expression, far more than expected by chance (p-value: <10^−10^, Fisher's exact test, Figure S4). The rate of YBX1/signature gene co-expression was even higher among the 151 genes (74%, corresponding to 111 genes) recovered in the set of matched tumor/normal specimens. Moreover, we found a significant overrepresentation of U0126-responsive genes within the group of up-regulated genes in tumors with high YBX1 expression (193 genes, p-value: <10^−10^, Fisher's exact test). Furthermore, we observed a significant positive correlation of YBX1 expression with 28 of its target genes identified by ChIP-on-chip (Figure 8C, Figures S4 and S5, Table 1). Thirteen YBX1 targets identified in colorectal cancer cells by chromatin immunoprecipitation were also recovered in a previous study [37] (Table 1). This robust subset of YBX1 targets included *CCNB1*, supporting the crucial role of YBX1 in proliferation control in colorectal cancer.

Previous studies described the relationship between YBX1 expression and/or nuclear localization of the protein, phenotypic properties of cancer cells and cancer patient survival [39]–[46]. To assess the role of YBX1 as a prognostic factor in colorectal cancer, we analyzed data from tissue microarrays representing 103 primary colorectal cancers and 15 pulmonary metastases by immunohistochemistry using an YBX1-specific antibody (Figure 8D) [45]. We observed a trend that a higher cytoplasmic YBX1 expression in primary tumors was associated with worse prognosis, however, the log-rank test failed to reach statistical significance. Whereas the nuclear expression of YBX1 in primary tumors was not correlated with patient survival, the nuclear staining of pulmonary metastases of colorectal cancers was clearly associated with a worse prognosis, pinpointing the close relationship between YBX1 function, high proliferative potential and poor outcome of the disease. All statistical values are embedded in Figure 8E and the clinical data are shown in Table S7.

## Discussion

The RTK/RAS/MEK/ERK pathway transduces mitogenic stimuli and transformed phenotypes through profound alterations of the transcriptional program. To elucidate regulatory principles of pathway-triggered gene transcription, we firstly identified common clusters of target genes sensitive to MEK inhibition in three colorectal cancer cell lines carrying *KRAS* or *BRAF* mutations. Then we screened target gene promoters for predominantly represented transcription factor binding sites. By computational analysis we predicted E2F transcription factors and nuclear factor Y (NFY or CCAAT-binding factor, CBF) as potential regulators. Since the role of E2F in growth control is well established [47], we focused on elucidating the role of NFY-binding motifs, narrowed down the specific transcription factor binding to them and provided functional evidence for the role of YBX1 in controlling target gene expression downstream of MEK/ERK.

Several transcription factors can interact with NFY-binding sites and regulate diverse or identical targets (reviewed in [48]). For example, both Y-box binding protein 1 (YBX1) and NFY are known to be involved in regulating the *CCNB1* promoter [33]; [34]. Reporter assays confirmed that MEK/ERK-dependent stimulation of transcription is indeed mediated by NFY elements in the *CCNB1* promoter. Chromatin immunoprecipitation using NFY-A, NFY-B or YBX1 antibodies, respectively, revealed that YBX1 preferentially regulates *CCNB1* transcription in colorectal cancer cells. To further study the role of YBX1 in controlling MEK/ERK-dependent gene expression, we analyzed endogenous promoter sequences bound to YBX1 in HCT116 colorectal cancer cells by ChIP-on-chip assay. Besides *CCNB1* we recognized 62 genes within proliferation-associated gene clusters as YBX1 targets. This supported the notion that YBX1 is an important regulator of MEK/ERK-dependent proliferation-associated genes.

YBX1 belongs to the cold-shock domain (CSD) protein super-family and represents the most evolutionary conserved nucleic acid-binding protein currently known (for review see [49]). YBX1 exerts multiple functions including the regulation of transcription [50], translation [46]; [51], DNA repair [52], drug resistance [53], cellular invasion [46]; [54] and environmental stress response [55]. Several lines of evidence have indicated that YBX1 promotes proliferation. The YBX1 protein re-localizes from the cytoplasm into the nucleus in G1/S-phase of the cell cycle and stimulates expression of cyclin A, cyclin B1 and other targets [34]. Forced expression of *YBX1* in human mammary epithelial cells (HMEC) induced EGF-independent proliferation via activation of the EGFR pathway, presumably by direct transcriptional stimulation of *EGFR* gene expression rather than via autocrine stimulation [56]. Targeted disruption of *YBX1* alleles resulted in major defects in the G2/M phase [57], suppression of cell proliferation in cancer cells, associated with a reduction of cells in S-phase of the cell cycle [36], multi-organ hypoplasia and senescence in response to cellular stress [58]. *YBX1* transgene expression in mammary glands of lactating mice resulted in early onset of hyperplastic growth followed by progression to carcinomas [59]. Over-expression of *YBX1* in breast [53], prostate [60] and colon cancer [61] confirmed its role as a positive modulator of proliferation.

The cis-regulatory sequences of genes down-regulated on blocking the MEK/ERK pathway in colorectal cancer cells clearly differ from those of growth factor stimulated genes which for example harbor binding sites for the transcription factors ATF/CREB, NFκB and SRF [4]; [5]. While growth factor stimulation permitted the identification of primary effectors of the signaling cascade, our approach is more likely to identify primary and secondary consequences of a chronic RASMEK/ERK pathway activation. NFY-binding sites were not recovered in the promoters of growth factor-regulated genes. The MEK/ERK target gene clusters 13 and 23 only share binding sites for ELK and E2F transcription factors with the growth factor-regulated module. This suggests that the transcription factors ELK and E2F may be essential for both initiating and maintaining proliferative potential during transition from the quiescent to the proliferative state [47], while YBX1 serves a different function in growth control. The diversity of growth factor-stimulated effects and MEK/ERK-dependent effects in continuously cycling cells was also reflected at the target gene expression level. We identified dual-specificity phosphatase (*DUSP6*) as the only factor commonly regulated by YBX1 and the set of transcription factors involved in growth factor-induced stimulation. DUSP6 is involved in feedback regulation of the MAPK pathway and is necessary to shape its biological activity regardless of its initial mode of activation [62].

Several lines of evidence have already indicated a close relationship between YBX1 function and the RTK/RAS pathway including ERK and AKT, the downstream effectors of RAS. YBX1 cooperated with the AKT pathway to transform mammary epithelial cells [63]. YBX1 was identified as a direct target of RSK1 and RSK2, a group of kinases downstream of ERK [64]. YBX1 can also be phosphorylated directly via ERK and by GSK3β [65]. Notably, treatment of basal-like breast cancer cells with the MEK inhibitor PD98059 resulted in the inhibition of YBX1 function [65] and the knock-down of YBX1 in breast cancer cells harboring activated RAS resulted in growth suppression [66]. The effects of the MEK inhibitor U0126, which completely blocked MEK/ERK activity, and the growth inhibition observed in the HCT116 cell line following the silencing of *YBX1* expression support the notion that the effects on YBX1 are likely to be mediated by ERK and/or RSK1/2 in colorectal cancer cells.

High *YBX1* expression and/or nuclear localization are closely associated with poor prognosis in several types of cancer [39]–[44]. Therefore, we sought to define a YBX1-related target gene signature in colorectal cancer. About 29% of the genes in proliferation- associated clusters are up-regulated in primary colorectal cancer samples compared to matched normal tissue, demonstrating the relevance of the MEK/ERK-dependent expression signatures and the YBX1 regulator for colorectal cancer biology. The expression of YBX1 in primary colorectal carcinomas correlated well with the expression of YBX1 target genes including *CCNB1*, which are involved in cell cycle control. Higher levels of nuclear YBX1 expression in pulmonary metastasis were associated with poor outcome in colorectal cancer patients. We assume that this is due to highly proliferative metastatic cells.

In contrast to the consequences of the MEK inhibitor U0126, the effects of the sulindac metabolites are more difficult to understand. These inhibitors interfere with RAS signaling, however, are not sufficient to block MEK/ERK during a period of 48 hrs. Alterations of the CRAF phosphorylation status after sulindac metabolite treatment point at cellular feedback mechanisms that may be responsible for sustained MAPK activity. The observation that the two sulindac metabolites affect growth effect in HCT116 and SW480 cells indicates that additional effectors of the signalling system may contribute to the overall proliferative potential.

In summary, we have combined signaling interference, transcriptomic profiling and computational analysis of cis-regulatory elements of target genes to identify YBX1 as a transcriptional regulator downstream of the mitogen-activated protein kinase (MEK/ERK) pathway. The results reported in this paper prove the feasibility of utilizing pathway-restricted gene expression data and computational analysis of genomic DNA adjacent to transcriptional start sites for elucidating regulatory principles. Moreover, the data define necessary future studies to close the gap between understanding the role of a single factor in controlling deregulated gene expression in cancer cells and the comprehension of gene regulation at the systems level. Specific pathway inhibitors, neutralizing antibodies and siRNAs are useful tools for dissecting networks in further detail, particularly for linking groups of transcriptional targets (or modules) and defined branches of signaling pathways. Several layers of complexity will have to be further analyzed, presumably at the level of individual transcription factor targets. Future work will have to address the combinatorial effects of E2F and YBX1/NFY transcription factors [30]; [67], the dual role of YBX1 in controlling transcription and translation of mRNA targets, the role of YBX1 phopshorylation through ERK2 and AKT [51]; [63]; [65], and the biological function in regulating cellular growth in cell lines, tumors and metastases.

## Methods

### Cell lines and cell culture

The colon carcinoma cell lines SW480, HT29 and HCT116 were cultured in complete L15 medium at 37°C and 5% CO_2_ in a humidified incubator. To block RAS-mediated signal transduction the following inhibitors, dissolved in DMSO (final concentration: 0.29%), were added to the medium for 48 h at the indicated concentrations: EGFR inhibitor AG 1478 (300 nM), RAS/RAF interaction inhibitors sulindac sulfide (100 µM) and sulindac sulfone (200 µM; all Merck Biosciences GmbH, Schwalbach, Germany), MEK inhibitors PD098059 (16 µM; Alexis Deutschland GmbH, Grünberg, Germany) and U0126 (20 µM; Promega GmbH, Mannheim, Germany). Control cultures were adjusted to the same DMSO concentration as inhibitor-treated cells. Following treatments, cells were directly lysed in a buffer containing 1% SDS, 10 mM Tris/HCl, pH = 7.5, and 2 mM EDTA.

### Proliferation assays

One thousand cells were seeded in 96-well plates and incubated with medium containing inhibitors or DMSO only for 24–72 hr. Growth of cells was determined in triplicate experiments by a colorimetric XTT-based assay (Roche Diagnostics GmbH, Mannheim, Germany). The XTT reagent was prepared and added to cells in 24 h intervals according to the manufacturer's protocol. After 4 h of incubation we determined the extinction at 480 nm. All extinction measurements were calculated relative to the DMSO-control after 72 hr. The means of at least three independent experiments are presented.

### Immunoblots

Ten µg of whole cell lysates per lane were fractionated by SDS-PAGE and blotted onto nitrocellulose membranes (Schleicher and Schuell, Dassel, Germany). The following antibodies were used for specific protein detection: cyclin B1, ERK2, α-CBFA (NFYB) and α-CBFB (NFYA) (Santa Cruz Biotechnology, Santa Cruz, USA), P-C-RAF, P-MEK, P-ERK (Cell Signalling Technology, Beverly, USA), ERK1 (BD Biosciences, Heidelberg, Germany), YBX1 (peptide specific N- and C-terminal antibodies). Secondary antibodies coupled to horseradish peroxidase were used in combination with the ECL detection system (Amersham Biosciences, Freiburg, Germany).

### Cell cycle analysis

To determine the cell cycle distribution of inhibitor-treated cells, both adherent and floating cells were collected, washed twice in phosphate-buffered saline, and fixed in 70% ethanol. Samples were stored at 4°C. Prior to flow cytometry, samples were centrifuged, the supernatants were discarded and the remaining pellets suspended in dilution buffer (0.1% Triton X-100, 0.5% BSA in phosphate buffered saline) supplemented with 80 µg/ml DNase-free RNase (Roche Diagnostics, Mannheim, Germany). Samples were incubated at room temperature for 30 min. RNase was removed by centrifugation and cell pellets were stained with 20 µg/ml propidium-iodide (Fluka, Heidelberg) in dilution buffer. Cell suspensions were analyzed by flow cytometry on a FACS Calibur system (BD Biosciences, Heidelberg, Germany). Single cell populations were gated from the received dot blots using WinMDI software (V. 2.8; Joseph Trotter; freeware) and the resulting histograms further analyzed for cell cycle distribution with using Cylchred software (V. 1.0.0.1; UWCM). All measurements were performed in duplicate.

### Microarray analysis

The HG-U133A human oligonucleotide microarray (Affymetrix, Santa Clara, CA, USA) comprises 22,283 known genes. Labeling of RNA targets, hybridization and post-hybridization procedures were performed according to protocols provided by Affymetrix. Following washing and staining, probe arrays were scanned twice at 3 µm resolution using a confocal argon laser scanner (Hewlett-Packard, Santa Clara, CA), controlled by Microarray Suite 5.0 software (Affymetrix). Photoemission was detected by a photomultiplier tube through a 570-nm long-pass filter. Computer-generated array images were overlaid with a virtual grid, controlled by Microarray Suite 5.0 software. This step allowed definition of each feature and alignment within known array dimensions. About 40 pixels within each feature were averaged after discarding outliers and pixels close to feature boundaries. Gene expression levels were calculated according to the average hybridization intensities of perfectly matched versus mismatched oligonucleotide probes. Arrays were scaled by Microarray Suite 5.0 software to an average hybridization intensity of 2,500 per gene and analyzed independently. The Data Mining Tool 3.0 (Affymetrix) and GeneSpring software package 6.1 (Silicon Genetics, Redwood City, CA) were used for comparing expression profiles of inhibitor-treated colorectal cancer cell lines and DMSO controls. The data were normalized to compensate for variability in hybridizations and hybridization artifacts.

### Validation of array expression data by quantitative real-time PCR

cDNAs of selected target genes were obtained by reverse transcription of 2 µg of total RNA using the High Capacity cDNA Archive Kit (Applied Biosystems, Foster City, USA). PCR amplification was done on TaqMan low density arrays using the ABI PRISM 7900HT Sequence Detection System (384-well format) according to the manufacturer's instructions (http://www.appliedbiosystems.com). Primers were synthesized by Applied Biosystems (Table 2). We used SDS2.2-analysis software (Applied Biosystems) for quantification. RNA prepared from DMSO-treated cells was used as the calibrator sample and GAPDH primers as endogenous control detector.

**Table 2:** Validation of gene expression by real-time RT-PCR.

Gene symbol	HG-U133A-ID	Taqman Assay-ID
*AURKB*	209464_at	Hs00177782_m1
*CCNB1*	214710_s_at	Hs00259126_m1
*CCNB2*	202705_at	Hs00270424_m1
*CDC2*	203213_at	Hs00176469_m1
*CDC25C*	205167_s_at	Hs00156411_m1
*CDK2*	204252_at	Hs00608082_m1
*CDKN3*	209714_s_at	Hs00193192_m1
*CHEK1*	205394_at	Hs00176236_m1
*CKS2*	204170_s_at	Hs00854958_g1
*HMMR*	209709_s_at	Hs00234864_m1
*MKI67*	212020_s_at	Hs00606991_m1
*PCNA*	201202_at	Hs00427214_g1
*Pfs2*	221521_s_at	Hs00211479_m1
*RFC2*	1053_at	Hs00267983_m1
*RFC3*	204127_at	Hs00161357_m1

15 genes initially identified in proliferation-associated clusters were selected for validation using Taqman Q-PCR. The table summarizes the gene symbols and the corresponding Affymetrix-IDs and Taqman-Assay-IDs.

### GO-analysis

To identify potential biological functions associated with gene clusters, we analyzed the Gene Ontology (GO) annotations of co-expressed genes. To avoid redundant sequence representations, we mapped the probe set identifiers of significantly detected probe sets and gene clusters to LocusLink identifiers. These were annotated with GO-terms using HomGL [68], available at http://homgl.gene-groups.net. To test the biological processes represented in Gene Ontology for significant overrepresentation in annotated gene clusters as compared to the set of all expressed genes, we applied the GOSSIP algorithm [69], available at http://gossip.gene-groups.net. We chose the significance threshold such that the false discovery rate was below 5%.

### In silico promoter analysis

Potential regulatory regions of all genes that entered the cluster analysis ranging from 1-kb upstream to the transcription start site of the longest transcript were extracted from the human genome database version NCBI35, Ensembl version 30 [70]. These regions were scanned for all 559 motifs of known transcription factor binding sites represented as positional frequency matrices in Transfac version 9.4 [71] using a published algorithm [72]. Overrepresentation of binding sites in clusters was determined by estimating the false discovery rate (FDR) using the hyper-geometric distribution as described [29]. A threshold of FDR <0.05 was applied and FDR was validated by running the same analyses on 100 randomly chosen clusters of the same size. All genes that entered the cluster analysis were used as a reference set.

### Transient transfection of *CCNB1* reporter genes

Chloramphenicol acetyl transferase (CAT) reporter plasmids harboring either the *CCNB1* promoter from nucleotides −57 to +182 (p240B1-CAT) or the same fragment with mutations in both NFY-binding sites (p240B1mPX-CAT [32]) were transiently transfected into HCT116 cells. The pCAT3-Basic vector and mock transfections served as controls. Transfer efficiency was controlled by transfection of pCAT-control, a reporter construct harboring the SV40 promoter (BD Biosciences, Heidelberg, Germany). The conditions for transfections and analysis of reporter gene activity by CAT enzyme linked immunosorbent assay were described previously [55]. To assess the effect of MAPK signal transduction on *CCNB1* promoter activity, transfected HCT116 cells were treated with 20 µM U0126 for 48 h. The amount of CAT protein in transfectants was normalized to the protein content of the corresponding cellular lysate and expressed as % CAT expression relative to that of p240B1-CAT transfections. Values are given as average of duplicate transfections from 2 independent experiments.

### Chromatin immunoprecipitation (ChIP)

The ChIP-assay was done as described with modifications [73]. In brief, 2×10^6^ cells were plated prior to U0126-treatment for 48 h. To compensate for the higher proliferation rate, 1×10^6^ cells treated with solvent only were plated in parallel. Following fixation and removal of the cytoplasm, nuclei were re-suspended in 600 µl nuclear lysis buffer (50 mM Tris/HCl, pH 8.0, 10 mM EDTA, 1% SDS and protease inhibitors). Two treated samples were combined and sonicated. After centrifugation, the supernatants were transferred into new tubes and the DNA content was determined by spectrophotometry at 260 nm. An equivalent of 100 µg DNA per sample was diluted 1∶10 in a buffer containing 20 mM Tris/HCl pH = 8.0, 150 mM NaCl, 2 mM EDTA, 1% Triton X100 and protease inhibitors and incubated with 4 µg of the following antibodies for 16 h: α-YBX1 (directed aginst a c-terminal peptide of YBX1) [74], α-CBFA (NFYB) (sc-7711x) and α-CBFB (NFYA) (sc-7712x) (both from Santa Cruz Biotechnology, Santa Cruz, USA), α-V5 (Invitrogen, Karlsruhe, Germany). Protein-A-agarose was added for 60 min to collect antibodies and bound DNA:protein complexes. The beads were washed 3-times with low salt buffer (20 mM Tris/HCl pH = 8.0, 150 mM NaCl, 2 mM EDTA, 1% Triton, 0,1%SDS), and once with high salt buffer (20 mM Tris/HCl pH = 8.0, 500 mM NaCl, 2 mM EDTA, 1% Triton, 0,1%SDS). The precipitated DNA:protein complexes were eluted by incubation with 0,1 M NaHCO_3_, and 1%SDS. NaCl was added to a final concentration of 200 mM and the samples were incubated at 65°C for 4 h. DNA was extracted with phenol/chloroform and precipitated using “Pelletpaint” for 16 h. The pellets were diluted in 40 µl pure water and 2,5 µl-aliquots were subjected to 35 cycles of PCR using an Advantage 2 system (BD Bioscience). The following primers were used for the amplification of promoter-specific DNA fragments: *CCNB1* (fragment –142 to +57): forward primer (5′-AGAGGCAGACCACGTGAGAG-3′), reverse primer (5′-GCCAGCCTAGCCTCAGATTTA-3′); *GAPDH* (fragment –908 to –603): forward primer (5′-GGATGGAATGAAAGGCACAC-3′), reverse primer (5′-GTTTCTGCACGGAAGGTCAC-3′).

For a genome-wide analysis of YBX1:DNA binding we took advantage of the NimbleGen array service including labeling of probes and array hybridization. Preparations for ChIP-on-chip probes from HCT116 cells were done according to NimbleGen protocols (supplied by RZPD, Berlin, Germany). Amplification of precipitated DNA and input control was carried out essentially as described [75] with one round of PCR amplification (20 cycles) followed by a second round (10 cycles). Amplified DNA was purified using the QIAquick PCR purification kit (Quiagen) according to the manufacturer's instructions. The raw hybridization intensities of each channel of the array (NimbleGen Homo sapiens HG17 promoter) were normalized and log-transformed using Bioconductor VSN package [76]. Fold changes were calculated by subtraction of Cy3 (input) from Cy5 intensity (ChIP sample). Normalized probe levels were smoothed along chromosomal coordinates using a sliding window method. For each probe position the smoothed probe level was computed as the median over the probe levels in an 800-bp window centered at that position. A cut-off was defined for enriched probes assuming a normal distribution of the smoothed data and calculating the 86% quantile. Enriched probes were merged into enriched regions, if less than 600-bp apart. Resulting regions of at least five probes were called enriched sites. The statistical significance of YBX1-ChIP target over-representation in clusters 10, 13 and 23 was determined by Fisher's exact test. A p-value below 0.05 was considered significant.

### Electrophorectic mobility shift assay

Nuclear extracts were prepared using the nuclear extraction kit (Pierce, Rockford, USA) according to the manufacturer's instructions. Three µg of each nuclear extract were used in a binding assay with 20 fmol of a biotin-labeled single-stranded oligonucleotide derived from the *CCNB1* promoter (5′-CTGGAAACG CATTCTCTGC GACCGGCAGCC GCCAATGGGA AGGGAGTGAG TGCCACGAAC-3′). For competition experiments a 100-fold excess of the same unlabelled oligonucleotide was added. The presence of YBX1 in the retarded DNA:protein complexes was confirmed by adding 2 µg of α-YBX1 N-terminal antibody or an unrelated control antibody α-CBFA (NFYB) (Santa Cruz Biotechnology, Inc., Santa Cruz, USA). The binding reactions were incubated at 30°C for 15 min before adding the labeled oligonucleotide. After further incubation of 30 min the samples were fractionated by electrophoresis through a 6% SDS-polyacrylamide gel. The biotin-labeled oligonucleotides were visualized after transfer onto a nylon membrane by incubation with streptavidin-horseradish peroxidase conjugate according to a standard protocol (Pierce, Rockford, USA).

### Analysis of MEK-dependent gene signatures and YBX1 expression in primary colon carcinomas and metastases

Expression data (obtained by interrogating Affymetrix HG-U95A microarrays) of primary colorectal carcinomas and matched normal colonic tissue were selected from a previously published study [38]. Differentially regulated genes were determined with the SAM algorithm (Significance analysis of microarrays; [77]) using the “samr” package [78] provided in the software environment “R” for statistical computing and graphics (version R 2.9, [79]). The delta value was set to obtain a false discovery rate (FDR) of <0.05 and the threshold of fold changes was set to ≥2.

Furthermore, we divided all primary colorectal cancers into two groups according to their YBX1 expression: tumours with YBX1 expression less than the mean (low expressing group); tumours with YBX1 expression higher than the mean (high expressing group). We identified differentially expressed genes using “samr” with a false discovery rate (FDR) of <0.05. The over-representation of U0126-responsive genes in tumours with high YBX1 expression was determined using Fisher's exact test. A p-value <0.05 was considered significant.

Pearson correlation coefficients of YBX1 expression and the expression of genes represented on the Affymetrix HG-U95A array were calculated for 43 primary colorectal cancers characterized in the same study using R 2.9. A p-value <0.05 was considered to indicate a significant correlation.

To be able to compare the expression data obtained from cell lines (analyzed by interrogating HG-U133A microarrays) with those from the tumor samples, we extracted a matched list of genes represented on both types of microarrays. A total of 532 genes responsive to U0126 treatment were also represented on the HG-U95A chip platform.

Immunohistochemical scores of cytoplasmic and nuclear YBX1 expression in primary colon carcinoma and pulmonary metastases and survival data were selected from a previous tissue microarray analysis [45]. Kaplan-Meyer curves were calculated and plotted using the package “survival” [80] in R (version 2.9). The design and processing of tissue microarrays was essentially as described previously [45].

### Silencing of YB-1 expression by siRNA

Specific siRNA against YB-1 (ID # 115541 and 115542) as well as an unrelated control siRNA (Silencer Negative control # 1 siRNA, Cat# 4611) were obtained from Ambion (Austin, USA). Two transfection systems were used in this study: a) HCT116 cells were plated and cultured for 24 h and then transfected twice in an interval of 24 h with the specific or control siRNA at a final concentration of 5 ηM using oligofectamine (data shown in Figure 6D). b) HCT116 cells were transfected using siPORT Amine according to the manufacturer's protocol (Ambion, Austin, USA) (data shown in Figure S2).

### Microarray data sets

Microarray data sets are available at http://www.ncbi.nlm.nih.gov/geo/under accession codes GSE18232 and GSE18337.

### Ethics statement

This study was conducted according to the principles expressed in the Declaration of Helsinki. The study was approved by the Institutional Review Board of the Robert-Rössle-Clinic, Berlin-Buch. All patients provided written informed consent for the collection of samples and subsequent analysis. This study does not include any animal experiments.

## Supporting Information

Figure S1 Inhibitor effects on oncogenic signaling pathways in colorectal cancer cells. (A) Western blot analysis of c-RAF phosphorylated at Ser256 (P-c-RAF), total c-RAF, P-MEK1/2 and P-ERK levels in colon carcinoma cells treated with the indicated inhibitors for 48 h. DMSO, solvent-only control. Total ERK and β-actin levels were determined to control for equal loading of cellular lysates. Heterogeneous effects of inhibitors other than the MEK-inihibitor U0126: PD098059 showed a partial reduction of phosphorylated ERK1/2 after 48 h and the other inhibitors had no effect. MEK phosphorylation was not significantly changed after this period of time, but there were distinct alterations in the c-RAF status. The sulindac metabolites caused an increase of c-RAF phosphorylation at Ser256 in SW480 and a partial increase in HT29 cells. Treatment of HCT116 cells with the same compounds resulted in two different forms of phosphorylated c-RAF. AG1478 and PD098059 showed weak effects, while the U0126 incubation resulted in a reduction of c-RAF phosphorylation in HT29 cells as well as in diminished RAF protein levels in HCT116 and SW480 cells, respectively. (B) Western blot analysis of P-AKT levels in colon carcinoma cells treated with the indicated inhibitors for 48 h. DMSO, solvent-only control. (loading control: total AKT levels).
(1.09 MB TIF)

Figure S2 Effects of YBX1 knock-down on cell growth and cell cycle distribution. (A) Proliferation of HCT116 cells was measured after transfection with YBX1 specific or scrambled siRNAs using the XTT assay. The treatment with transfection reagents alone is shown as mock control. Scr, scrambled siRNA (control). *, p-value: <0.025, single-sided T-test). (B) Proliferation of HCT116 cells (XTT assay) transfected with increasing amounts of scrambled or YBX1-specific siRNA. *, **, p-values: <0.025 and <0.0025, respectively. (C) Flow cytometric analysis of cell cycle distribution of HCT116 cells after siRNA transfection. For ease of comparison, bar diagram includes data obtained with the DMSO solvent control and U0126 treatment shown in Figure 3B.
(0.59 MB TIF)

Figure S3 Differentially expressed genes in four matched pairs of normal and tumor tissue as determined by SAM analysis. False discovery rate <0.05, fold change >2; red: up-regulated genes; green: down-regulated genes. The list of differentially expressed genes is shown in Table S6.
(0.22 MB TIF)

Figure S4 Correlation of MEK/ERK-dependent target gene and YBX1 expression in colorectal cancer. We generated 100 random sets of 151 (A), 523 (B) and 851 genes (C), respectively, from the genes represented on the Affymetrix HG-U95A microarray. The size of the gene sets corresponds to (A) the number of proliferation-associated genes up-regulated in four colon tumors relative to their matched normal tissues, (B) to the number of proliferation-associated genes identified in clusters 02, 10, 13, 23 and (C) to all genes up-regulated in four colon tumors relative to their matched normal controls, respectively. For each set, we calculated the fraction of genes showing a significant positive or negative (discordant) correlation with YBX1 expression in the set of 43 primary colorectal cancers and displayed the frequencies graphically. The red dots mark the fraction of positive or negative correlations in the experimentally defined sets.
(0.47 MB TIF)

Figure S5 Coexpression of YBX1 and YBX1-target genes. Supplement to Figure 8C. Further examples of YBX1 and YBX1 target co-expression in 43 primary colorectal tumors as detected on Affymetrix HG-U95A microarrays. YBX1 targets shown in (A) were also identified in basal-like breast cancer cells (Ref. 37; Finkbeiner, M.R., Astanehe, A., To, K., Fotovati, A., Davies, A.H., Zhao, Y., Jiang, H., Stratford, A.L., Shadeo, A., Boccaccio, C., Comoglio, P., Mertens, P.R., Eirew, P., Raouf, A., Eaves, C.J., and Dunn, S.E. [2009]. Profiling YB-1 target genes uncovers a new mechanism for met receptor regulation in normal and malignant human mammary cells. Oncogene 28: 1421–1431). (B) Novel YBX1 targets. Pearson correlation coefficients (coef) and p-values are inserted in the graphs.
(0.35 MB TIF)

Table S1 Normalized expression values obtained by microarray analysis of colorectal cancer cell lines treated with inhibitors. Columns indicate the probe set-ID on Affymetrix HG-U95A microarrays, gene symbol, number of cluster (see Figure 3) and the normalized expression values of all 18 experiments with 3 cell lines, 5 inhibitor treatments and DMSO (solvent) control.
(2.09 MB XLS)

Table S2 Statistical features of gene clustering were obtained by calculating the distance of each gene to the center of the cluster (parameter: in) and the distance to the center of the closest other cluster (parameter: out). The fit coefficient was computed using the formula: abs (out minus in)/(out plus in).
(1.75 MB XLS)

Table S3 Table of genes in U0126-responsive clusters. Affymetrix probe-set ids, gene symbols and normalized expression of genes in cluster 02, 10, 13 and 23.
(2.04 MB XLS)

Table S4 List of overrepresented Gene Ontology (GO) terms related to the products of clustered target genes. Columns indicate the cluster number, GO-identifier, GO-term and false discovery rate (FDR) calculated by the GOSSIP algorithm (Ref. 69; Blüthgen, N., Brand, K., Cajavec, B., Swat, M., Herzel, H., and Beule, D. [2005]. Biological profiling of gene groups utilizing gene ontology. Genome Inform 16: 106–115). In addition, the last four columns show the number of genes in each cluster corresponding to the GO-term, the number of genes with the GO-term not present in the cluster, genes without the GO-term in the cluster, and all genes without the GO-term, not present in the cluster.
(0.04 MB XLS)

Table S5 Prediction of transcription factor binding sites. The procedure for identifying transcription factor binding sites in the regulatory sequences of MEK/ERK pathway-regulated genes is described in Materials and Methods. Columns indicate probe set-IDs, gene symbols, the number of the cluster of co-expressed genes and the predicted binding sites for NFY, E2F and HOX4A (marked by ***).
(1.88 MB XLS)

Table S6 Differential gene expression in four colorectal cancers and matched normal tissues. SAM analysis of microarray data (Affymetrix HG-U95A), FDR <0.05, fold change >2.
(0.23 MB XLS)

Table S7 Clinical data of tissue microarray study. Data collection and description of technique: Ref. 45; Knösel T, Emde A, Schlüns K, Chen Y, Jürchott K, Krause M, Dietel M, Petersen I. (2005). Immunoprofiles of 11 biomarkers using tissue microarrays identify prognostic subgroups in colorectal cancer. Neoplasia. 7:741–747. Columns of table: T, TNM stage; N, nodal status; M, metastasis, 0, no metastasis, 1, metastatic tumor; G, grade. Immunohistochemical detection of YBX1 protein in cytoplasm and nucleus; 0, no staining; 1, weak, 2 intermediate, 3, strong staining (see Figure 8D for an example). Death: 0, patient alive at the time of analysis; 1, patient deceased. Survival time indicated in weeks.
(0.03 MB XLS)
