# Supplementary figures and images for: Identification of Y-Box Binding Protein 1 As a Core Regulator of MEK/ERK Pathway-Dependent Gene Signatures in Colorectal Cancer Cells
Source: PLoS Genet. 2010 Dec 2;6(12):e1001231. doi: 10.1371/journal.pgen.1001231 (PMC2996331; doi:10.1371/journal.pgen.1001231)

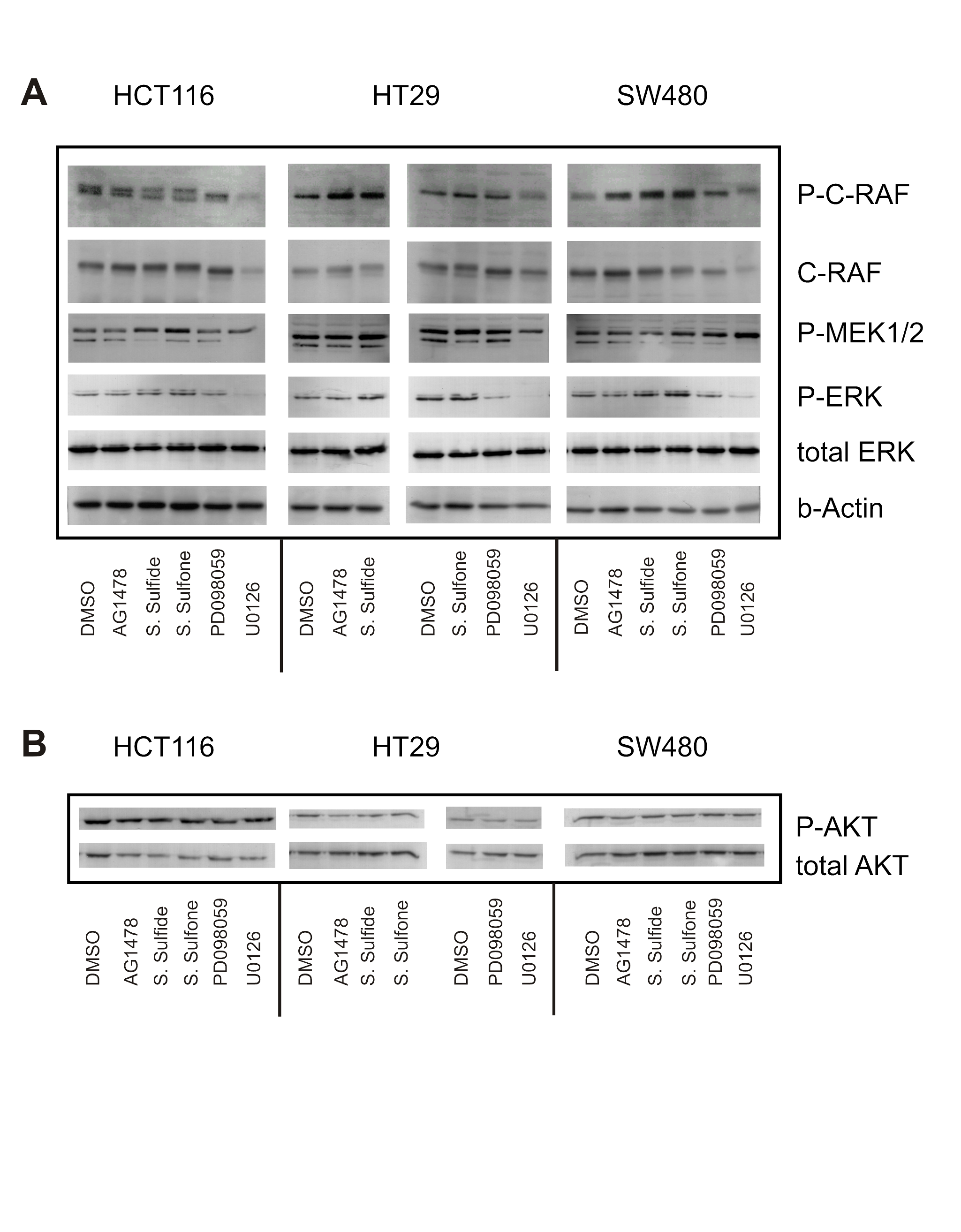

Supplement: Figure S1 — Inhibitor effects on oncogenic signaling pathways in colorectal cancer cells. (A) Western blot analysis of c-RAF phosphorylated at Ser256 (P-c-RAF), total c-RAF, P-MEK1/2 and P-ERK levels in colon carcinoma cells treated with the indicated inhibitors for 48 h. DMSO, solvent-only control. Total ERK and β-actin levels were determined to control for equal loading of cellular lysates. Heterogeneous effects of inhibitors other than the MEK-inihibitor U0126: PD098059 showed a partial reduction of phosphorylated ERK1/2 after 48 h and the other inhibitors had no effect. MEK phosphorylation was not significantly changed after this period of time, but there were distinct alterations in the c-RAF status. The sulindac metabolites caused an increase of c-RAF phosphorylation at Ser256 in SW480 and a partial increase in HT29 cells. Treatment of HCT116 cells with the same compounds resulted in two different forms of phosphorylated c-RAF. AG1478 and PD098059 showed weak effects, while the U0126 incubation resulted in a reduction of c-RAF phosphorylation in HT29 cells as well as in diminished RAF protein levels in HCT116 and SW480 cells, respectively. (B) Western blot analysis of P-AKT levels in colon carcinoma cells treated with the indicated inhibitors for 48 h. DMSO, solvent-only control. (loading control: total AKT levels). (1.09 MB TIF) [file pgen.1001231.s001.tif]

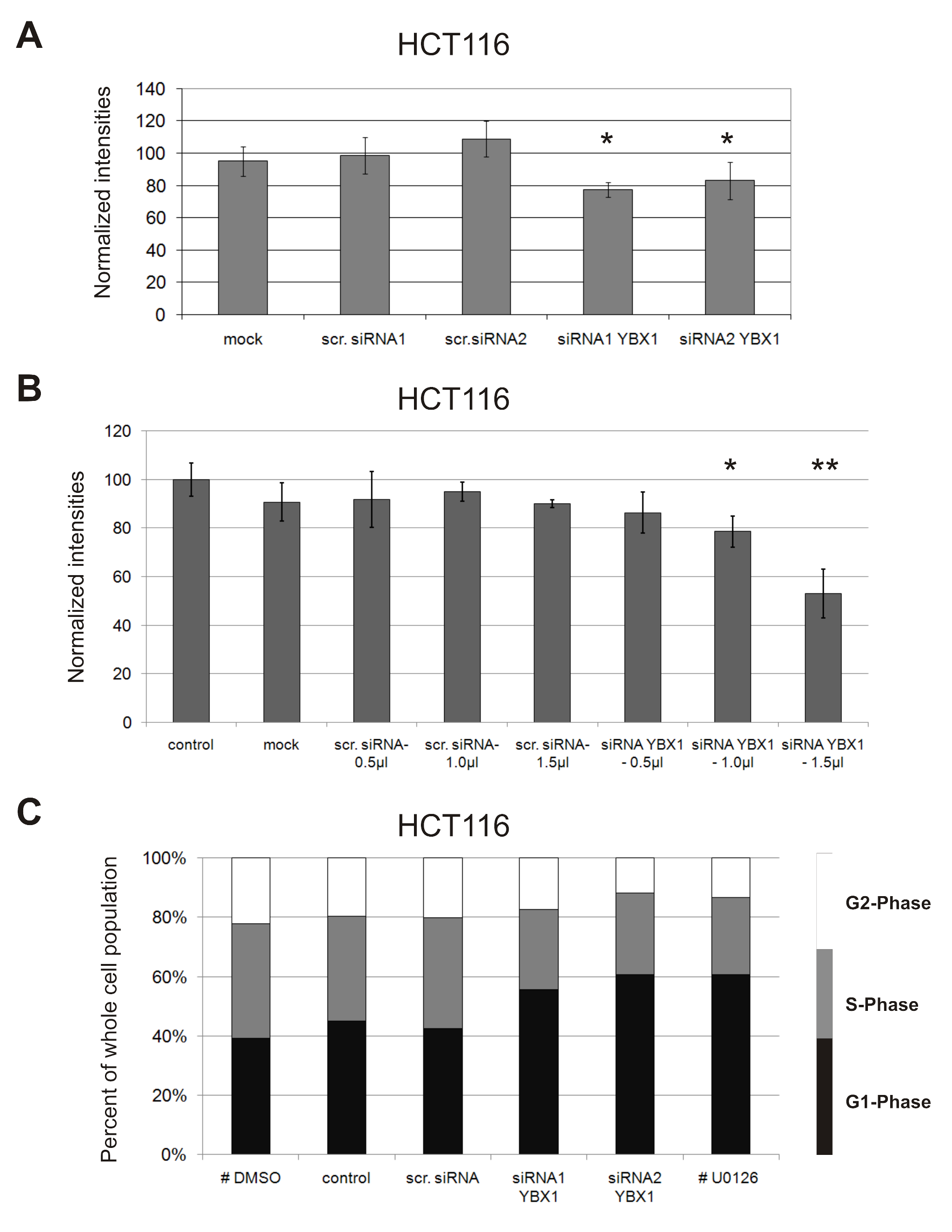

Supplement: Figure S2 — Effects of YBX1 knock-down on cell growth and cell cycle distribution. (A) Proliferation of HCT116 cells was measured after transfection with YBX1 specific or scrambled siRNAs using the XTT assay. The treatment with transfection reagents alone is shown as mock control. Scr, scrambled siRNA (control). *, p-value: <0.025, single-sided T-test). (B) Proliferation of HCT116 cells (XTT assay) transfected with increasing amounts of scrambled or YBX1-specific siRNA. *, **, p-values: <0.025 and <0.0025, respectively. (C) Flow cytometric analysis of cell cycle distribution of HCT116 cells after siRNA transfection. For ease of comparison, bar diagram includes data obtained with the DMSO solvent control and U0126 treatment shown in Figure 3B. (0.59 MB TIF) [file pgen.1001231.s002.tif]

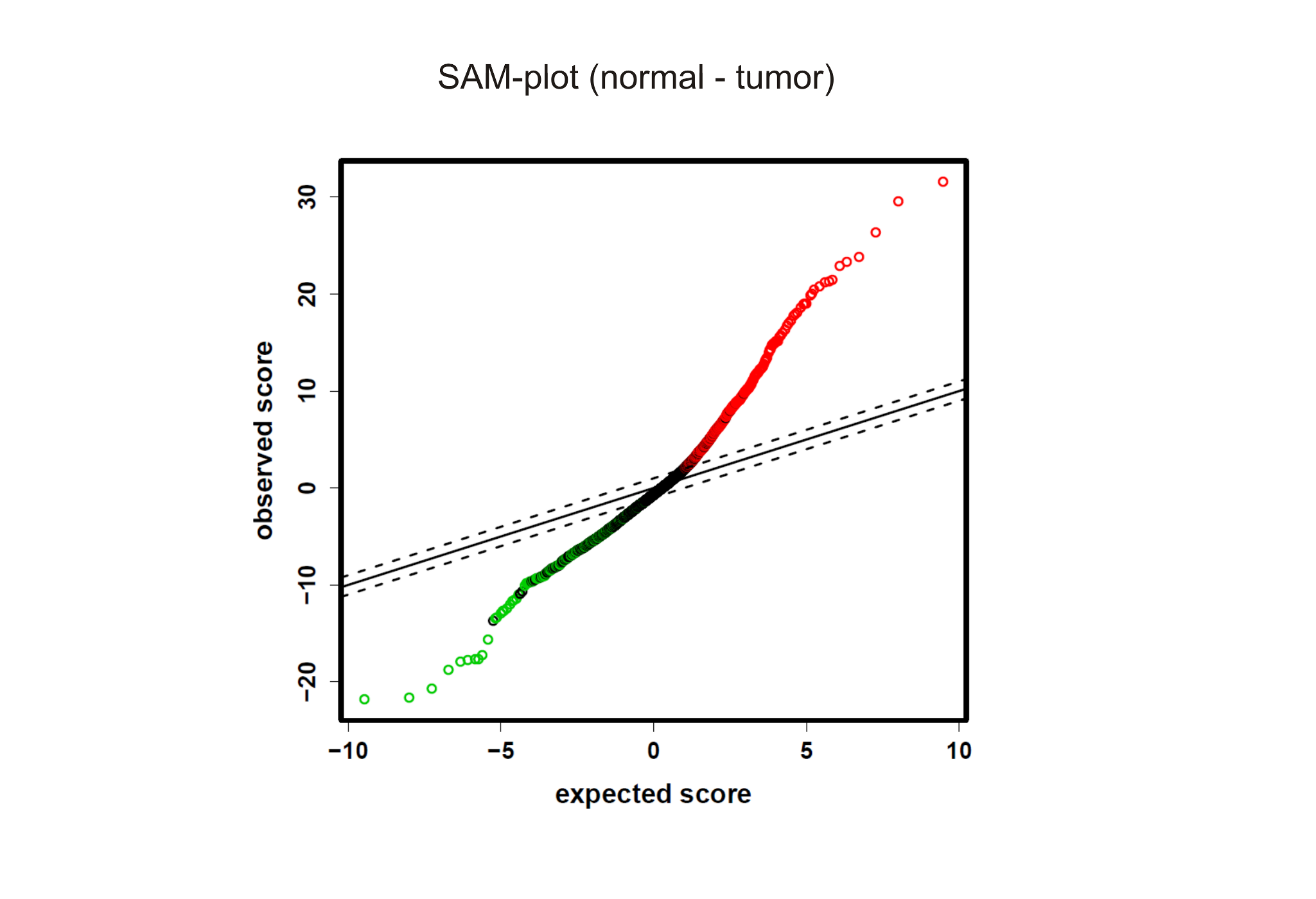

Supplement: Figure S3 — Differentially expressed genes in four matched pairs of normal and tumor tissue as determined by SAM analysis. False discovery rate <0.05, fold change >2; red: up-regulated genes; green: down-regulated genes. The list of differentially expressed genes is shown in Table S6. (0.22 MB TIF) [file pgen.1001231.s003.tif]

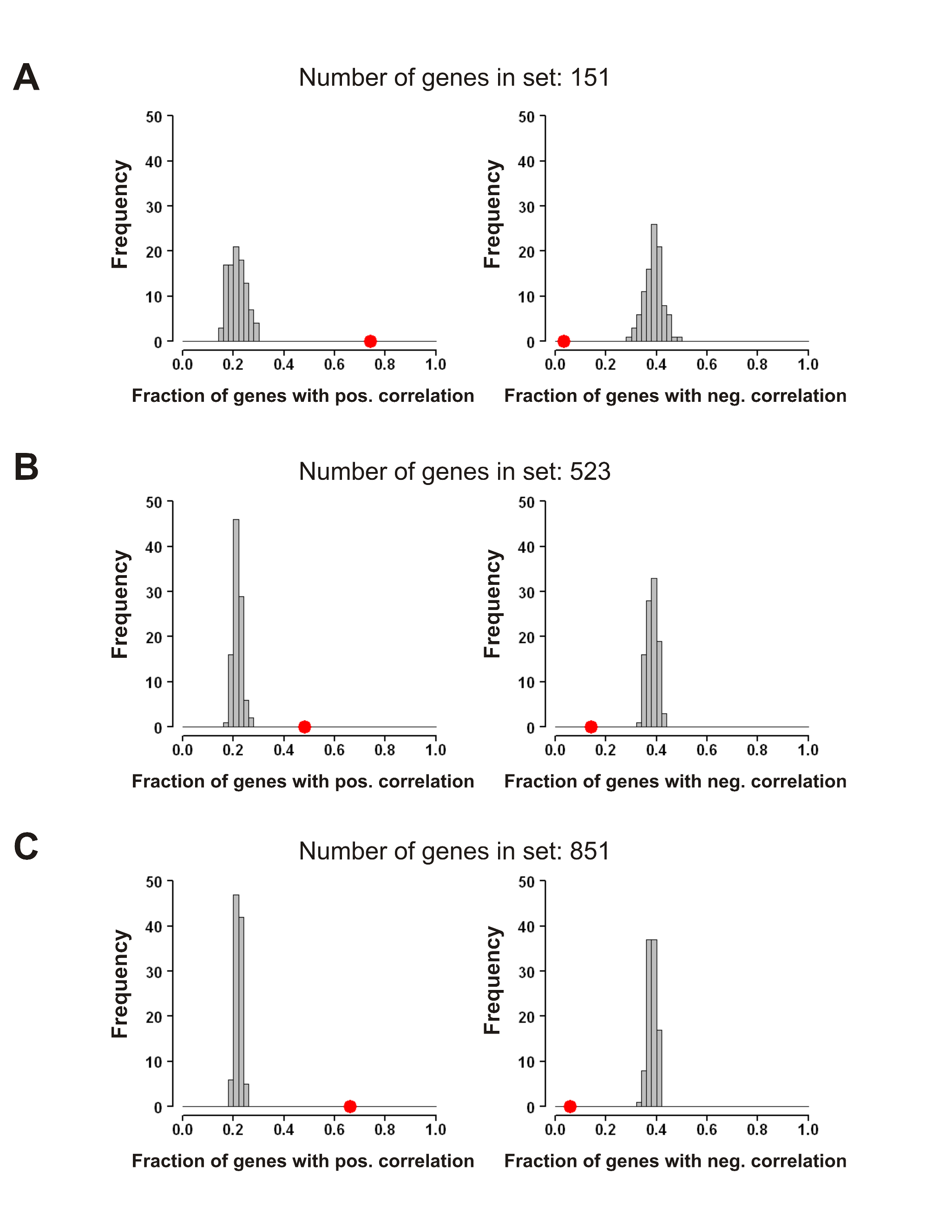

Supplement: Figure S4 — Correlation of MEK/ERK-dependent target gene and YBX1 expression in colorectal cancer. We generated 100 random sets of 151 (A), 523 (B) and 851 genes (C), respectively, from the genes represented on the Affymetrix HG-U95A microarray. The size of the gene sets corresponds to (A) the number of proliferation-associated genes up-regulated in four colon tumors relative to their matched normal tissues, (B) to the number of proliferation-associated genes identified in clusters 02, 10, 13, 23 and (C) to all genes up-regulated in four colon tumors relative to their matched normal controls, respectively. For each set, we calculated the fraction of genes showing a significant positive or negative (discordant) correlation with YBX1 expression in the set of 43 primary colorectal cancers and displayed the frequencies graphically. The red dots mark the fraction of positive or negative correlations in the experimentally defined sets. (0.47 MB TIF) [file pgen.1001231.s004.tif]

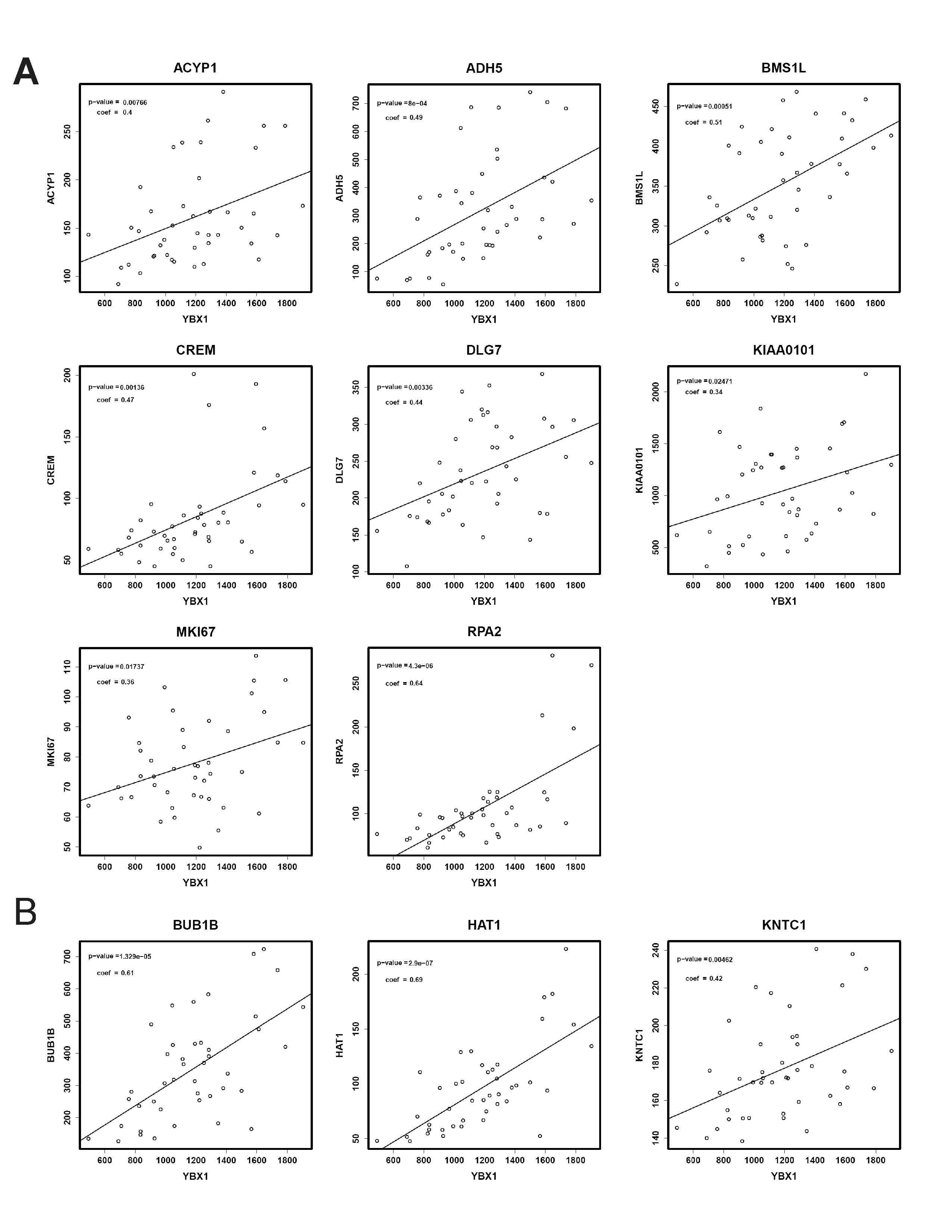

Supplement: Figure S5 — Coexpression of YBX1 and YBX1-target genes. Supplement to Figure 8C. Further examples of YBX1 and YBX1 target co-expression in 43 primary colorectal tumors as detected on Affymetrix HG-U95A microarrays. YBX1 targets shown in (A) were also identified in basal-like breast cancer cells (Ref. 37; Finkbeiner, M.R., Astanehe, A., To, K., Fotovati, A., Davies, A.H., Zhao, Y., Jiang, H., Stratford, A.L., Shadeo, A., Boccaccio, C., Comoglio, P., Mertens, P.R., Eirew, P., Raouf, A., Eaves, C.J., and Dunn, S.E. [2009]. Profiling YB-1 target genes uncovers a new mechanism for met receptor regulation in normal and malignant human mammary cells. Oncogene 28: 1421–1431). (B) Novel YBX1 targets. Pearson correlation coefficients (coef) and p-values are inserted in the graphs. (0.35 MB TIF) [file pgen.1001231.s005.tif]
